# Supramolecular Engineering of Nanoceria for Management and Amelioration of Age‐Related Macular Degeneration via the Two‐Level Blocking of Oxidative Stress and Inflammation

**DOI:** 10.1002/advs.202408436

**Published:** 2025-01-10

**Authors:** Mingyu Xu, Yifan Zhou, Yufeng Xu, An Shao, Haijie Han, Juan Ye

**Affiliations:** ^1^ Eye Center, The Second Affiliated Hospital School of Medicine Zhejiang Provincial Key Laboratory of Ophthalmology Zhejiang Provincial Clinical Research Center for Eye Diseases Zhejiang Provincial Engineering Institute on Eye Diseases Zhejiang University 88 Jiefang Road Hangzhou 310009 China

**Keywords:** age‐related macular degeneration, host–guest interaction, inflammation, nanoceria, oxidative stress

## Abstract

Age‐related macular degeneration (AMD), characterized by choroidal neovascularization (CNV), is the global leading cause of irreversible blindness. Current first‐line therapeutics, vascular endothelial growth factor (VEGF) antagonists, often yield incomplete and suboptimal vision improvement, necessitating the exploration of novel and efficacious therapeutic approaches. Herein, a supramolecular engineering strategy to construct moringin (MOR) loaded α‐cyclodextrin (α‐CD) coated nanoceria (M@CCNP) is constructed, where the hydroxy and newly formed carbonyl groups of α‐CD interact with the nanoceria surface via O─Ce conjunction and the isothiocyanate group of MOR inserts deeply into the α‐CD cavity via host–guest interaction. By exploiting the recycling reactive oxygen species (ROS) scavenging capability of nanoceria and the anti‐inflammation properties of MOR, the two‐level strike during AMD pathogenesis can be precisely blocked by M@CCNP. Remarkably, excellent therapeutic efficacy to CNV is observed in vivo, achieving over 80% reduction in neovascularization and over 60% reduction in leakage area. In summary, the supramolecular engineered nanoceria provides an efficient approach for amelioration of AMD by blocking the two‐level strike, and presents significant potential as an exceptional drug delivery platform, particularly for ROS‐related diseases.

## Graphical abstract

This study reports a supramolecular engineering strategy to construct moringin loaded α‐cyclodextrin coated nanoceria (M@CCNP) for the treatment of age‐related macular degeneration (AMD). By exploiting the recycling reactive oxygen species (ROS) scavenging capability of nanoceria and the anti‐inflammation properties of moringin, the two‐level strike during AMD pathogenesis can be precisely blocked by M@CCNP.

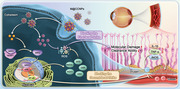

## 1 Introduction

As the primary cause of irreversible vision loss worldwide, age‐related macular degeneration (AMD) imposes a substantial and continuously growing economic burden on society.^[^ ^1^ ^]^ The vascular endothelial growth factor (VEGF) inhibition strategy remains the first‐line treatment, and its corresponding products (pegaptanib, ranibizumab, and aflibercept) have been approved for AMD treatment by the U.S. Food and Drug Administration (FDA).^[^ ^2^ ^]^ However, the repeated and life‐lasting administration, the adverse reaction of macular dysfunction after long‐term use of VEGF antagonists, the 25–40% response rate, and the high cost of the commercial products urge the seeking of alternative targets for the treatment of AMD.^[^ ^3^ ^]^

The two‐level strike model shifts the focus from the downstream VEGF signaling pathway to the upstream pathogenesis of AMD.^[^ ^4^ ^]^ Initially, within the aging framework, excessive oxidative stress and impaired clearance ability lead to the accumulation of molecular damage at the Bruch's membrane, culminating in the formation of drusen. While drusen formation is a hallmark of AMD, it is not solely sufficient to drive neovascularization. Subsequently, the inflammatory host response is triggered, leading to irreversible retinal dysfunction and activation of the downstream VEGF signaling pathway. Moreover, these two processes can mutually exacerbate each other, forming a vicious cycle that accelerates VEGF production. Elevated VEGF levels, in turn, promote choroidal neovascularization (CNV), a late‐stage pathological manifestation of AMD and the direct cause of irreversible blindness.^[^ ^5^ ^]^ Thus, therapeutic strategies targeting these two strikes may contribute to a higher response rate and improved therapeutic effect.

Nanoceria, as potent auto‐regenerative antioxidant nanoparticles, possess unique properties of reversibly switching oxidation states between Ce^3+^ and Ce^4+^. The renewable valence states enable nanoceria to exhibit multienzyme mimetic activities, including superoxide dismutase (SOD), catalase (CAT), oxidase, and phosphatase.^[^ ^6^ ^]^ The auto‐regenerative anti‐oxidative properties, combined with its easy surface modification, superior size‐dependent penetrability, and efficient cellular internalization,^[^ ^7^ ^]^ have significantly advanced the biomedical applications of nanoceria.^[^ ^8^ ^]^ The therapeutic potential of nanoceria has been extensively studied and confirmed in various diseases affecting different systems or organs, particularly in ocular diseases.^[^ ^9^ ^]^ Moringin (MOR), a hydrophobic isothiocyanate compound, has been reported to have potent anti‐inflammatory properties by strongly activating NRF2 and inhibiting the NF‐κB pathway,^[^ ^10^ ^]^ and has demonstrated remarkable therapeutic potential across a wide range of inflammatory diseases.^[^ ^11^ ^]^ Considering the critical role of inflammation in AMD pathogenesis, MOR presents significant therapeutic potential, but its hydrophobicity and low bioavailability may limit its efficacy in treating AMD.

Cyclodextrin, as an FDA‐approved biocompatible additive, is widely used for surface modification due to its unique truncated cone‐like 3D structure with a hydrophilic exterior and a hydrophobic cavity, which enables the formation of inclusion complexes via host–guest interactions.^[^ ^12^ ^]^ Interestingly, α‐cyclodextrin (α‐CD) could smartly load MOR via the host–guest interaction to form MOR/α‐CD inclusion complex, thus improving the solubility and bioavailability of MOR.^[^ ^13^ ^]^ More importantly, α‐CD can be coated on the surface of nanoceria due to its feasibility of surface modification,^[^ ^14^ ^]^ making it an ideal bridge between nanoceria and MOR.

**Figure 1:**
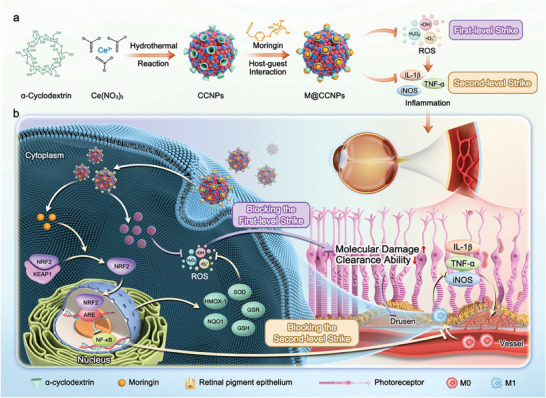
Schematic illustration of M@CCNP for management and amelioration of AMD via blocking the two‐level strike. a) The synthesis of M@CCNP by one‐pot hydrothermal method for CCNP preparation and host–guest interaction for MOR loading. b) The underlying therapeutic mechanism of M@CCNP via its unique properties of auto‐regenerative ROS scavenging and inflammation suppression, thus blocking the two‐level pathological response of AMD.

In this work, we reported MOR‐loaded α‐cyclodextrin (α‐CD) coated nanoceria (M@CCNP) for blocking the two‐level strike of AMD. A one‐pot hydrothermal method strategy was employed to achieve the successful α‐CD surface coating of the nanoceria (**Figure** **1**a). Apart from providing enhanced drug delivery potential, α‐CD coating contributed to lowering toxicity and increasing the water solubility of the bare nanoceria. Subsequently, MOR was loaded onto the CCNP via host–guest integration. Upon intravitreal injection, the small size, high water solubility, and neutral potential of M@CCNP facilitated its accumulation in the lesion sites at the fundus. At the lesion sites, nanoceria effectively blocked the first‐level strike of oxidative stress by eliminating newly generated reactive oxygen species (ROS) and preventing the further triggering of inflammatory responses. Particularly, the reversibly switching oxidation states of nanoceria contributed to the regenerative and continuous therapeutic efficacy of M@CCNP. Concurrently, MOR was expected to block the existing second‐level strike, exerting its effects via the activation of the nuclear factor erythroid 2‐related factor 2 (NRF2), therefore inhibiting the M1 activation of macrophages through the NRF2/NF‐κB signaling pathway (Figure 1b).^[^ ^10^, ^15^ ^]^ Altogether, our supramolecular‐engineered nanoceria provides a prospective therapeutic strategy for the management and amelioration of AMD as well as exploiting the drug delivery potential of nanoceria.

## 2 Results

### 2.1 Synthesis and Characterization of M@CCNP

**Figure 2:**
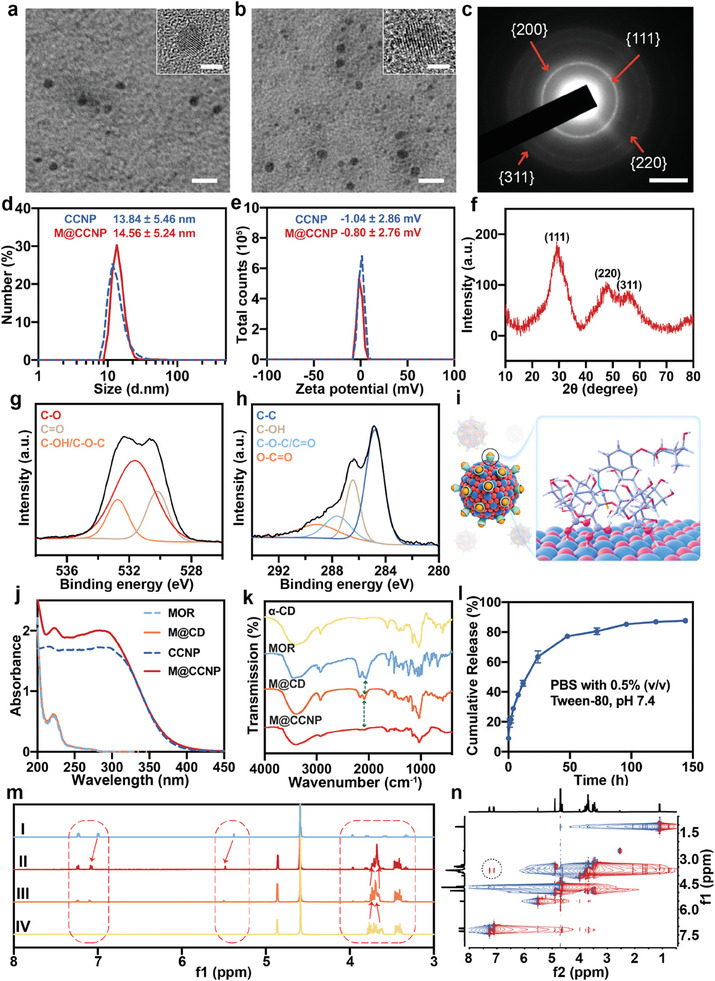
Preparation and characterizations of the nanoceria. Representative TEM and HRTEM images of CCNP (a) and M@CCNP (b); scale bar = 10 nm/2.5 nm (insert). c) SAED images of M@CCNP, scale bar = 5 nm^−1^. Hydrodynamic diameters (d) and Zeta potentials (e) of CCNP and M@CCNP. (f) XRD pattern of M@CCNP. XPS analysis of O 1s (g) and C 1s (h) of CCNP. (i) The simulated surface structure of M@CCNP. j) UV–vis absorbance spectra of MOR, M@CD, CCNP, and M@CCNP. (k) FTIR spectra of α‐CD, MOR, M@CD, and M@CCNP. Green dashed line: the displacement of the characteristic peak of the isothiocyanate group from 2054.88 cm^−1^ (free MOR) to 2086.83 cm^−1^ (M@CD)/2086.12 cm^−1^ (M@CCNP). (l) In vitro release profile of MOR from M@CCNP in releasing medium (PBS with 0.5% (v/v) Tween‐80, pH 7.4). Results are presented as mean ± SD; n = 3. (m) ^1^H‐NMR spectra of (I) 16 mM MOR, (II) 16 mM MOR/16 mM α‐CD, (III) 5.3 mM MOR/16 mM α‐CD, (IV) 16 mM α‐CD. (n) 2D NOESY spectrum of M@CD.

The synthetic route of the M@CCNP was illustrated in Figure 1a, encompassing the following steps. Initially, α‐CD coated nanoceria (CCNP) was prepared via a one‐pot hydrothermal method.^[^ ^14^ ^]^ Subsequently, MOR was loaded onto the CCNP through the host–guest interaction between the α‐CD and MOR. As shown in the transmission electron microscopic (TEM) images and high‐resolution TEM (HRTEM) images, both CCNP (**Figure** **2**a) and M@CCNP (Figure 2b) displayed uniform, finely dispersed, and near‐spherical morphology with 2–4 nm in diameter. The dynamic light scattering (DLS) analysis revealed similar hydrodynamic diameters for CCNP and M@CCNP, measuring 13.84 ± 5.46 and 14.56 ± 5.24 nm (Figure 2d), along with near‐neutral Zeta potentials of −1.04 ± 2.86 and −0.80 ± 2.76 mV, respectively (Figure 2e). The small size offered a larger surface area compared with larger particles, thereby ensuring a higher number of defects and oxygen vacancies and higher efficiency of ROS adsorption during an autocatalytic reaction. Besides, the small and near‐spherical morphology facilitated efficient penetration of the viscous vitreous humor and inner limiting membrane (ILM) (an average pore size of 10–25 nm).^[^ ^16^ ^]^ Additionally, the near‐neutral potential promoted unhindered diffusion within the vitreous humor. The typical fluorite cubic structure of the M@CCNP was confirmed by the X‐ray diffraction (XRD) analysis and the selected area electron diffraction (SAED) analysis (Figure 2c,f).

The successful coating of α‐CD onto the nanoceria was confirmed by Fourier transform infrared (FTIR) spectra analysis, where the spectra of CCNP exhibited the characteristic peaks of α‐CD at 1155.10 (C─O) and 1030.40 (C─O─C) cm^−1^, and a narrower hydroxy band (3392.08 cm^−1^) than the original α‐CD (Figure 2k; Figure S1, Supporting Information).^[^ ^17^ ^]^ The XPS survey of O 1s and C 1s spectra of CCNP provided further insight into the surface interaction between α‐CD and nanoceria. Three peaks at 532.7 (C─OH/C─O─C), 531.6 (C─O), and 530.2 (C═O) eV were observed in O 1s spectra, while four peaks at 284.8 (C─C), 286.4 (C─OH), 287.6 (C─O─C/C═O), and 289.1 (O─C═O) eV were observed in C 1s spectra (Figure 2g,h). In comparison with the O 1s and C 1s spectra of α‐CD (Figures S2 and S3, Supporting Information), the presence of carboxyl groups in CCNP was newly detected, accompanied by the decrease of hydroxy groups, which is akin to the findings in β‐CD modified gold nanoparticles (β‐CD@AuNPs).^[^ ^12^ ^]^ It assumed that the oxidation of hydroxy groups contributed to the controlled oxidation of Ce^3+^ to Ce^4+^, and the resulting carboxyl groups hindered the unlimited growth of the nanoceria via O─Ce conjunction, allowing nanoceria to grow in an orderly, controlled manner and ultimately presenting a small‐sized, nearly spherical morphology. Verification of MOR loading and host–guest molecular recognition was conducted via UV–vis spectra, FTIR spectra, ^1^H‐Nuclear Magnetic Resonance (^1^H‐NMR) spectra, and 2D nuclear overhauser effect spectrum (NOESY). UV–vis spectra indicated the presence of the characteristic peak of MOR at 228 nm in both MOR/α‐CD inclusion complexes (M@CD) and M@CCNP solution, signifying successful MOR loading (Figure 2j). FTIR spectra demonstrated the displacement of the characteristic peak of the isothiocyanate group to higher wavenumbers with reduced absorbance (from 2054.88 cm^−1^ of free MOR to 2086.83 cm^−1^ of M@CD/2086.12 cm^−1^ of M@CCNP), corroborating the occurrence of the inclusion complexes (Figure 2k). Furthermore, ^1^H‐NMR spectra revealed upfield shifts occurred for the protons on MOR, accompanied by upfield and downfield shifts of the H3 and H5 on α‐CD, respectively, which are located inside the cavity of α‐CD (Figure 2m). Nuclear Overhauser effect correlations were detected between the H3 protons of α‐CD and protons of MOR in the 2D NOESY spectrum, which strongly supported the successful guest molecule encapsulation (Figure 2n). Molecular dynamics simulations were further conducted to elucidate the surface structure and interaction mode of M@CCNP. As shown in Figure 2i, within a single α‐CD, two carboxyl groups oxidizing from hydroxy groups and one hydroxy group interact with the surface of nanoceria via O─Ce conjunction. Between MOR and α‐CD, the isothiocyanate group of MOR deeply inserts into the α‐CD cavity while the Rhamnose residue is exposed to the outside, aligned with the above experiments.

To elucidate the composition of the final product, The α‐CD composition was calculated by Anthrone spectrophotometric methods (Figure S4, Supporting Information),^[^ ^18^ ^]^ and the ceria composition was measured by inductively coupled plasma mass spectrometry (ICP‐MS) analysis. The actual MOR loading was measured according to the standard curve obtained for MOR using UV–vis spectrophotometer at 228 nm (Figure S5, Supporting Information). Consequently, the stoichiometry of each component was calculated as Ce: α‐CD: MOR = 43.91: 1.00: 0.78. Moreover, the excellent stability of CCNP was verified by no significant change in particle size for up to 4 weeks in PBS storage or lyophilization and rehydration (Figures S6 and S7, Supporting Information). The release profile elucidated the in vitro release behavior of MOR in M@CCNP, indicating a continuous release of MOR for up to 100 h at maximum (Figure 2l). This prolonged release, attributed to the host–guest interaction between MOR and α‐CD, allowed the sustained efficacy of MOR post‐administration.

### 2.2 Auto‐Regenerative ROS Scavenging Properties of CCNP and M@CCNP

**Figure 3:**
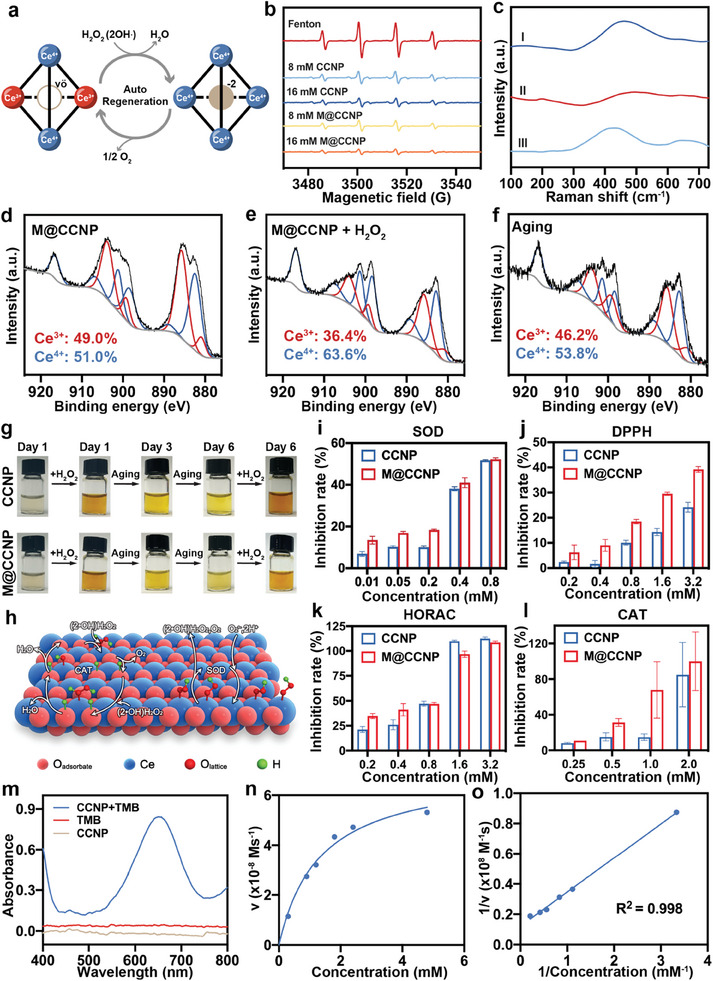
Auto‐regenerative ROS scavenging properties of CCNP and M@CCNP. a) Scheme of auto‐regenerative properties of nanoceria. b) ESR spectra after Fenton reaction with deionized water, 8 mM CCNP/M@CCNP, 16 mM CCNP/M@CCNP. c) Raman spectra of (I)M@CCNP, M@CCNP with the addition of H_2_O_2_ at (II) 0 min and (III) 60 min. XPS analysis of Ce 3d of fresh synthesized M@CCNP (d), M@CCNP + H_2_O_2_ at Day 0 (e) and Day 6 (f). g) Images of the color change of CCNP and M@CCNP after the reaction with H_2_O_2_ at varied time points. h) Scheme of enzyme‐mimic activity of nanoceria. i–l) The SOD‐mimic activity, DPPH scavenging ability, HORAC, and CAT‐mimic activity of CCNP and M@CCNP. m) UV–vis absorption spectra of the TMB‐CCNP system in sodium acetate buffer (pH 5.5). Michaelis‐Menten plot (n) and Lineweaver‐Burk plot (o) of the catalytic activity of CCNP in the TMB system. Results in (i–l) are presented as mean ± SD; n = 3.

The auto‐regenerative ROS scavenging properties of nanoceria, as illustrated in **Figure** **3**a, originate from the auto‐regenerative of Ce^3+^ from the Ce^4+^. The final product of nanoceria possessed a high percentage of Ce^3+^ quantified by X‐ray photoelectron spectroscopy (XPS), ensuring its ROS scavenging properties. Specifically, Ce^3+^ (885.7, 903.8, 899.2, 880.9 eV) constituted 49% of the total cerium, while Ce^4+^ (916.5, 882.4, 888.9, 898.4, 906.7, 901.1 eV) accounted for the remaining 51% of the total cerium (Figure 3d).^[^ ^19^ ^]^ Electron spin resonance (ESR) experiments were conducted to evaluate the hydroxyl radicals scavenging activity of the CCNP and M@CCNP. As shown in Figure 3b, the 1:2:2:1 multiple peak indicates DMPO–OH adducts created by the Fenton reaction, and the intensity of the peak represents the amount of free hydroxyl radicals. Both CCNP and M@CCNP exhibited a concentration‐dependent decrease in peak intensity.

To further assess the recyclable antioxidant properties of the final products, in situ Raman spectroscopy excited with a 488 nm laser recorded the spectra of M@CCNP during the oxidation reaction process (Figure 3c). The major peak centered at 460 cm^−1^ indicated a symmetric breathing mode of the oxygen atoms around Ce ions, reflecting ordering in the oxygen sublattice.^[^ ^20^ ^]^ The peak disappeared upon the addition of H_2_O_2_, signaling defect aggregation and sublattice disordering, but returned to its original intensity after 60 min, affirming the renewability of the nanoparticle. XPS spectra of M@CCNP and its oxidative products further confirmed the recyclability of the M@CCNP. As shown in Figure 3e,f, after H_2_O_2_ addition, Ce^3+^ oxide composition dropped by 12.6%, but restored to 46.2% within 6 days. Color changes, from light to dark yellow and back, visually depicted oxidation and renewal of both CCNP and M@CCNP (Figure 3g).

The surface presence of Ce^3+^/Ce^4+^, coupled with its self‐regenerative properties, endows nanoceria with multienzyme mimic ability, encompassing SOD‐mimic and CAT‐mimic activities (Figure 3h).^[^ ^21^ ^]^ Both CCNP and M@CCNP exhibited efficient decomposition of both radical (O_2_^−^• and OH•) and non‐radical (H_2_O_2_) oxidants in a concentration‐dependent manner, as verified by SOD (Figure 3i), 1,1‐Diphenyl‐2‐picrylhydrazyl radical 2,2‐Diphenyl‐1‐(2,4,6‐trinitrophenyl) hydrazyl (DPPH) (Figure 3j), hydroxyl radical antioxidant capacity (HORAC) (Figure 3k), and CAT assays (Figure 3l; Figure S8, Supporting Information).^[^ ^22^ ^]^ Besides, the loading of MOR amplified the multienzyme mimic ability of CCNP, especially at the low concentration, as evidenced by the higher inhibition rate or enzyme activity of the M@CCNP at a concentration below 0.4 mM. Furthermore, a steady‐state kinetic assay was performed to evaluate the oxidase‐mimic catalytic activity of CCNP. As expected, CCNP catalyzed the oxidation of colorless 3,3′,5,5′‐tetramethylbenzidine (TMB) into blue oxidized TMB (oxTMB), and the UV–vis spectra of the reaction products were shown in Figure 3m. The kinetic data were fitted to the Michaelis–Menten and Lineweaver–Burk models (Figure 3n,o), with the Michaelis‐Menten constant (K_m_) calculated to be 1.85 mM and the maximum velocity (V_max_) calculated as 8.23 × 10^−8^ M·s^−1^ of CCNP for TMB.

### 2.3 M@CCNP Suppressed In Vitro Oxidative Stress and Inflammation

Initially, we evaluated the biocompatibility and cellular uptake of M@CCNP to ensure its safety and effective accumulation at the fundus cells. The cell counting kit‐8 (CCK‐8) (Figures S9 and S10, Supporting Information) and live‐dead assays (Figures S11 and S12, Supporting Information) demonstrated the excellent biocompatibility of M@CCNP in macrophages (RAW264.7) and vascular endothelial cells (HUVEC). Furthermore, the results indicated that the nanoparticles were unlikely to suppress neovascularization via cytotoxicity. Subsequently, we investigated the cellular internalization of synthesized nanoceria using fluorescein as a labeling molecule, which can be encapsulated by α‐CD through host–guest interaction.^[^ ^23^ ^]^ Rapid and effective cellular uptake of CCNP was confirmed by both fluorescent images and flow cytometry analysis. Specifically, after Fluorescein@CCNP incubation, significant fluorescence signals were detected in RAW264.7 in a time‐dependent manner, reaching ≈50% intensity within an hour and maximal intensity at 2 h (Figures S13–15, Supporting Information). We further evaluated the cellular uptake mechanism of M@CCNP by comparing the cellular uptake of Fluorescein@CCNP at 37 °C and 4 °C after 2 h of incubation (Figure S16 and S17, Supporting Information). The results showed a significant reduction in fluorescence intensity at 4 °C, with a 66.1% decrease compared to the control, indicating that M@CCNP was actively uptaken by macrophages. Moreover, to investigate whether the uptake is mediated through endocytosis, we pretreated macrophages with inhibitors of different endocytic pathways, including chlorpromazine (CPZ, inhibitor of clathrin‐mediated endocytosis), amiloride (AML, inhibitor of macropinocytosis‐mediated endocytosis), and genistein (GEN, inhibitor of caveolin‐mediated endocytosis) before Fluorescein@CCNP incubation (Figures S16 and S17, Supporting Information). The fluorescence intensity decreased by 53.9%, 39.7%, and 26.5%, respectively, suggesting these three mechanisms, especially clathrin‐mediated endocytosis, contributed to the cellular uptake of M@CCNP. Overall, M@CCNP exhibited excellent biocompatibility and enhanced internalization mediated by endocytosis.

**Figure 4:**
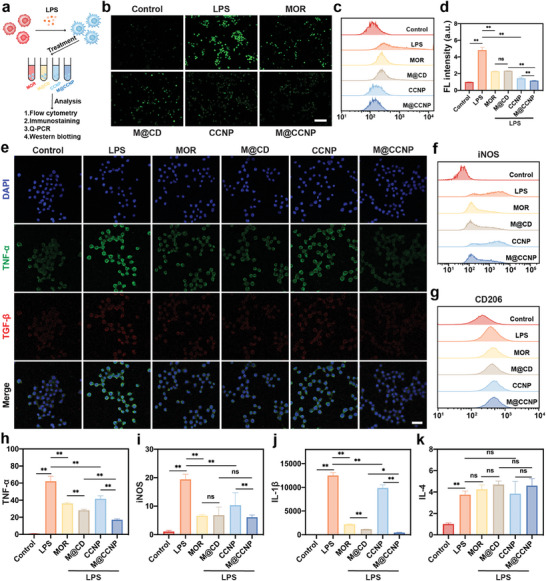
The intracellular anti‐ROS and anti‐inflammatory properties of M@CCNP in RAW264.7. (a) Schematic illustration of the experimental procedures to evaluate the intracellular ROS and inflammation with the treatment of MOR, M@CD, CCNP, and M@CCNP (MOR: 3.55 µM, CCNP: 200 µM). Representative fluorescent images (b) and flow cytometric curves (c) reflecting intracellular ROS level of RAW264.7 with different treatments using DCFH‐DA staining, and the corresponding quantitative results recorded by flow cytometry (d); scale bar = 100 µm. e) Representative confocal fluorescent images of RAW264.7 intracellular TNF‐α and TGF‐β expression after varied treatments. Green, TNF‐α; red, TGF‐β; blue, DAPI; scale bar = 25 µm. f,g) Flow cytometric curve of PE‐conjugated‐iNOS and APC‐conjugated‐CD206 fluorescent signals of RAW264.7 after different treatments. h–k) Q‐PCR analysis of TNF‐α, iNOS, IL‐1β, and IL‐4. Results in (d, h‐k) are presented as mean ± SD; n ≥ 3; ns, *p* > 0.05; *p < 0.05; **p < 0.01.

Within the two‐level strike framework, macrophages are the major source of ROS and inflammation in the fundus, and play a crucial role in the transition from a healthy fundus to age‐related macular dystrophy and eventually neovascular AMD.^[^ ^4^ ^]^ Thus, we evaluated the intracellular ROS scavenging and inflammation regulation ability of the M@CCNP in vitro macrophage models with lipopolysaccharide (LPS) as an inducer of excessive ROS and inflammatory state (**Figure** **4**a).

Intracellular ROS levels were assessed using the 2′′,7′′‐ dichlorofluorescein diacetate (DCFH‐DA) assay. Colorless DCFH‐DA can be oxidized to fluorescent 2′,7′‐dichlorofluorescein (DCF), with fluorescence intensity positively correlated to the ROS level. As shown in fluorescent images and flow cytometric analysis (Figure 4b–d; Figure S18, Supporting Information), excessive ROS in RAW264.7 cells were successfully induced by LPS compared with the control group, and all treatments, including free MOR, M@CD, CCNP, and M@CCNP, exhibited ROS inhibition effects. As shown in Figure 4d, among these groups, CCNP (decreased by 70.1%) and M@CCNP (decreased by 76.1%) displayed stronger inhibition ability than MOR (decreased by 52.9%) and M@CD (decreased by 51.6%), suggesting that nanoceria played the major role in ROS elimination.

Upon activation, blood‐derived monocytes can infiltrate the fundus and differentiate into either pro‐inflammatory or anti‐inflammatory subsets, secreting related cytokines.^[^ ^24^ ^]^ Among them, M1 macrophages are pro‐inflammatory and secrete multiple inflammatory mediators, including interleukin‐1β (IL‐1β), tumor necrosis factor‐α (TNF‐α), inducible nitric oxide synthase (iNOS), *etc*. On the contrary, M2 macrophages are anti‐inflammatory and secrete anti‐inflammatory cytokines. However, as the repair subset, M2 macrophages also secrete VEGF, which promotes angiogenesis.^[^ ^25^ ^]^ Hence, an optimal therapeutic approach for second‐level inflammation should aim at suppressing M1 polarization while avoiding the promotion of the M2 subset to prevent excessive secretion of VEGF. This strategy ensures a controlled inflammatory response and mitigates the risk of undesirable angiogenic effects.

The assessment of macrophage polarization and related cytokines were conducted to evaluate the anti‐inflammatory effects of M@CCNP. Flow cytometry analysis quantified both the M1 subset (marked by iNOS) and the M2 subset (marked by CD206) after LPS stimulation (Figure 4f,g; Figure S19, Supporting Information). The MOR‐containing treatments (MOR, M@CD, and M@CCNP) achieved significant inhibition of M1 polarization (decreased by more than 60%), while CCNP presented moderate inhibition effects (decreased by 34.9%). Compared with the LPS group, all four treatments showed no significant upregulation of the M2 subset. Consequently, M@CCNP showed great potential in inhibiting M1 polarization without increasing the M2 subset of the macrophages, further verified by fluorescent images stained with TNF‐α (M1 marker) and transforming growth factor‐β (TGF‐β, M2 marker) (Figure 4e; Figure S20, Supporting Information).

The expression of related cytokines was evaluated to verify the inflammatory regulatory effects of the M@CCNP. TNF‐α, iNOS, and IL‐1β are considered the pro‐inflammatory cytokines and markers of the M1 subset, and interleukin‐4 (IL‐4) is an anti‐inflammatory cytokine and inducer of the M2 subset. The quantitative results of Q‐PCR analysis were consistent with flow cytometry analysis (Figure 4h–k). In detail, MOR and M@CD exhibited stronger inhibition effects than CCNP, and M@CCNP presented the strongest downregulation of pro‐inflammatory cytokines. Particularly for TNF‐α and IL‐1β expression, a significant decrease was observed in M@CCNP group compared with M@CD and CCNP groups, suggesting the potential synergistic anti‐inflammatory effects of MOR and nanoceria. The strongest inhibition was observed on IL‐1β, reaching more than 96.3% in the M@CCNP group. Nonetheless, no obvious regulation of IL‐4 expression was observed after all four treatments.

As expected, M@CCNP exhibited excellent potential in inhibiting ROS and inflammation, which are the two major pathological processes of the two‐level strike pathogenesis. Specifically, MOR played a major role in alleviating inflammation via M1 inhibition, while nanoceria mainly contributed to ROS scavenging.

### 2.4 M@CCNP Activated NRF2 Signaling Pathway

**Figure 5:**
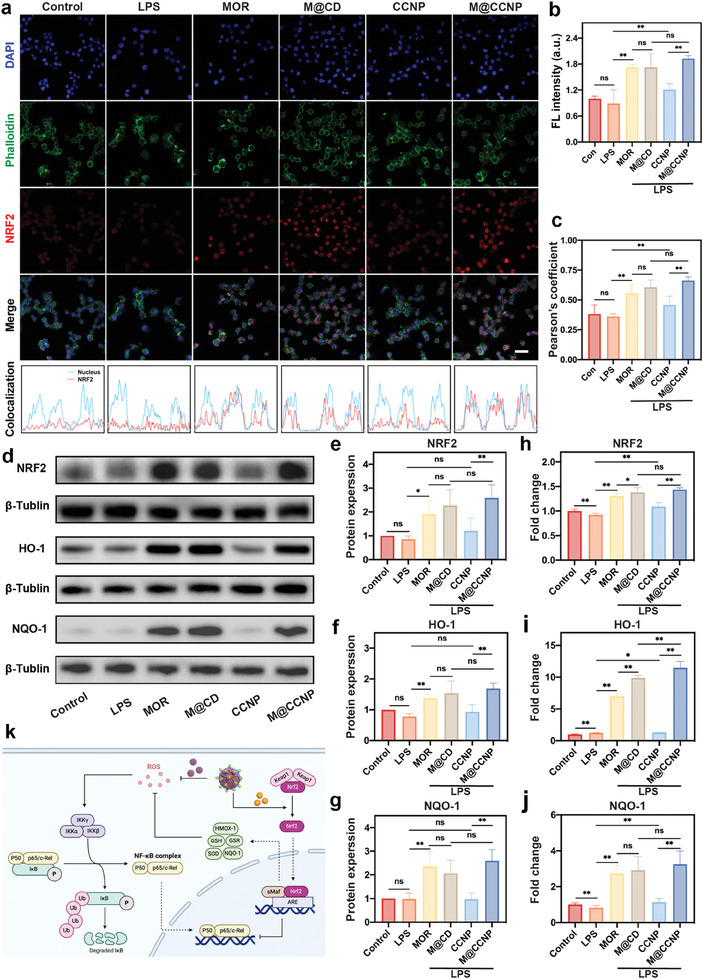
M@CCNP exerts its anti‐inflammatory effects via the activation of the NRF2 signaling pathway. a) The confocal fluorescent images of intracellular NRF2 expression (up) and colocalization analysis (down) with the treatment of MOR, M@CD, CCNP, and M@CCNP (MOR: 3.55 µM, CCNP: 200 µM). Green, Phalloidin; red, NRF2; blue, DAPI; scale bar = 25 µm. b,c) Quantitative results of NRF2 expression and Pearson's correlation coefficient of the colocalization between NRF2 and nucleus according to the fluorescent images. d) Western blotting images of NRF2, HO‐1, and NQO‐1 protein expression, and e–g) the corresponding quantitative results. h–j) Q‐PCR analysis of NRF2, HO‐1, and NQO‐1. k) Scheme of the intracellular mechanism of M@CCNP exerting its ROS and inflammation suppression properties. Results in (b‐c, e‐j) are presented as mean ± SD; n ≥ 3; ns, *p* > 0.05; *p < 0.05; **p < 0.01.

As demonstrated by the aforementioned experiments, M@CCNP exhibited excellent dual inhibition effects on ROS and inflammation. Among the two components, CCNP functioned through the auto‐recycling of the Ce^3+^/Ce^4+^ pattern. However, the underlying anti‐inflammatory and anti‐oxidative mechanism of MOR was not fully understood. NRF2 plays an indispensable role in inflammatory and ROS responses, regulating the macrophage polarization via the NRF2/NF‐κB signaling pathway and suppressing oxidative stress via the activation of antioxidant enzymes, including HO‐1, NQO‐1, etc (**Figure** **5**k). Therefore, we hypothesized that MOR could act as an NRF2 activator, thereby inhibiting downstream inflammation and oxidative stress.

As illustrated in the fluorescent images (Figure 5a,b), Q‐PCR (Figure 5h–j), and Western blotting (Figure 5d–g), both NRF2 and its downstream proteins HO‐1 and NQO‐1 were remarkably upregulated after MOR‐containing treatments. Apart from the increased expression, upon activation, the nuclear factor NRF2 separates from its binding protein Kelch‐like ECH‐associated protein 1 (KEAP1) and translocates from the cytoplasm to the nucleus to exert its anti‐inflammatory and anti‐oxidative functions. After treatment with MOR‐containing formulations, this translocation process was clearly visualized and verified by the colocalization curves of the nucleus and NRF2 signals, as illustrated in Figure 5a. The colocalization results were further quantified by Pearson's correlation coefficient, and the M@CCNP group demonstrated the most consistent distribution between the NRF2 and the nucleus (λ = 0.66) compared to the low consistency in the LPS group (λ = 0.36) (Figure 5c). CCNP group showed a slight increase in the expression of NRF2 and downstream proteins, and the difference was not always statistically significant, implying the limited NRF2 activation ability of nanoceria. In summary, MOR can act as an NRF2 activator and may regulate inflammation and ROS processes via NRF2 activation.

### 2.5 M@CCNP Alleviated Neovascularization and Leakage in Laser‐Induced CNV Mice

Given M@CCNP's demonstrated excellent inhibitive potential in the two‐level strike in vitro, along with its good biocompatibility and efficient cellular uptake, we proceeded to assess its therapeutic efficacy and safety in laser‐induced CNV mice, a classic animal model of neovascular AMD.

First, we evaluated the in vivo safety of the nanoparticles at both ocular and systematic levels in normal healthy mice and CNV mice. The normal mice received intravitreal injections of M@CCNP on Day 0, and short‐term and long‐term safety assessments were conducted on Day 5 and Day 28, respectively. At the ocular level, both short‐term and long‐term intraocular pressure (IOP) remained within the normal range and showed no significant difference from baseline IOP (Figure S21, Supporting Information). The scotopic electroretinography (ERG) tests revealed that both α and β waves were comparable to those of normal mice (Figure S22) and ocular histological analysis demonstrated no significant structural abnormalities or damage of the fundus at both Day 5 and Day 28 (Figure S23, Supporting Information), indicating M@CCNP has no significant acute or chronic toxicity to retinal function and structure. For systemic safety evaluation, blood samples were assessed with hematology (Figure S24, Supporting Information) and biochemistry (Figure S25, Supporting Information), and the parameters of all three groups were within the normal range. Major organs, including the heart, lung, liver, kidney, and spleen, underwent histological analysis and exhibited no structural abnormalities or signs of inflammation (Figure S23, Supporting Information). Besides, treated mice maintained stable body weight growth over 28 days, and there were no significant differences compared to the normal group (Figure S26, Supporting Information). For CNV mice, as revealed in normal mice, no significant ocular or systemic toxicity was detected as well (Figures S27–S30, Supporting Information). In summary, no ocular or systemic toxicity was observed in short‐term or long‐term safety evaluation or pathogenic conditions.

Then, the distribution and retention of M@CCNP in the fundus were investigated. The retention of nanoceria in the fundus was analyzed by ICP‐MS. As shown in Figure S31 (Supporting Information), Ce exhibited significant accumulation in the fundus at 6 h with the content of 14.4 ng per mg tissue, followed by a decrease by 24 h, after which its levels stabilized over the 1–7 days period. At Day 7, 7.84 ng mg^−1^ tissue of Ce remained in the fundus, demonstrating that nanoceria can persist in the fundus for an extended period. We further assessed the biodistribution of MOR in the fundus, with fluorescein as a surrogate since both can be encapsulated into CCNP by a‐CD via host–guest interaction and presenting comparable in vitro release behaviors (Figure 2l; Figure S32, Supporting information). As shown in Figure S33 (Supporting Information), green fluorescence was primarily accumulated in the inner layer of the retina at 3 h post‐injection, and significantly increased with even distribution throughout all layers at 6 h. By 12 h post‐injection, the fluorescence signal had significantly decreased, but still persisted by 48 h compared with the baseline. It can be inferred that MOR may rapidly accumulate in the fundus, followed by a significant clearance during the next several hours and a slower clearance thereafter.

**Figure 6:**
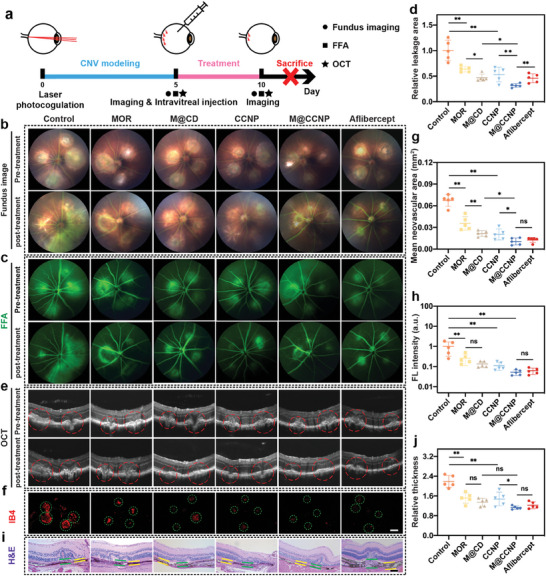
The in vivo therapeutic efficacy of M@CCNP. (a) Schematic illustration of the CNV modeling and treatment procedure. The corresponding fundus image (b), FFA (c), and OCT images (e) before and after the treatment of 2 µL of MOR, M@CD, CCNP, and M@CCNP (MOR: 17.8 µM, CCNP: 1 mM). (d) Quantitative calculation of the relative leakage area of different groups compared with the control group according to the FFA images. (f) The isolectin‐B4 immunostained choroidal flat mounts from each group, and the corresponding quantitative calculation of neovascular area (g) and integrated fluorescent intensity (h); scale bar = 250 µm. (i) The H&E staining image from each group, and (j) the quantitative analysis of relative CNV lesion. Green, the thickness of the CNV lesion site; yellow, the thickness of the normal choroid; scale bar = 100 µm. Results in (b, g, h, j) are presented as mean ± SD; n = 5; ns, *p* > 0.05; *p < 0.05; **p < 0.01.

Subsequently, we assessed the in vivo therapeutic efficacy of the nanoparticles. As illustrated in **Figure** **6**a, successful modeling was confirmed at 5 days after photocoagulation, followed by treating the laser‐induced CNV mice with intravitreal injections of saline (control group), MOR, M@CD, CCNP, M@CCNP, and aflibercept. Therapeutic efficacy was evaluated using fundus images, fundus fluorescein angiography (FFA), and optical coherence tomography (OCT) at both Day 5 and 10 of the same eye in each group. First, we analyzed the therapeutic efficacy among MOR, M@CD, CCNP, and M@CCNP groups. Notably, after saline administration, lesion expansion, and fusion were evident at the lesion site in fundus images (Figure 6b), along with enlarged areas of hyperfluorescent leakage in FFA images (Figure 6c). In contrast, all treatment groups showed obvious alleviation of the lesion and reduction of the leakage area. Specifically, the M@CD group exhibited less severe leakage than the MOR group, with M@CCNP showing the best therapeutic efficacy, further supported by quantitative calculation of leakage area reduction from FFA images (Figure 6d). Meanwhile, OCT images indicated reduced choroidal layer thickness at the lesion site and improved fundus structure integrity after treatment, and the most evident recovery was seen in the M@CCNP group (Figure 6e). The choroidal flat mounts of the CNV mice with different treatments were collected for further analysis, and the reduction of neovascularization area and florescent intensity was observed in IB4 stained choroidal flat mounts (Figure 6f–h). M@CCNP group exhibited the highest reduction rate of neovascularization area for more than 80%, and M@CD demonstrated superior performance compared to the free MOR group, which may be attributed to the enhanced stability and sustained release of MOR achieved by its positive interaction with α‐CD. Additionally, the relative thickness of lesion site choroidal tissues compared to surrounding normal choroidal tissues was calculated from H&E staining slides in each group, and the M@CCNP group displayed the highest reduction rate of the relative thickness (Figure 6i,j). To benchmark the therapeutic effects of M@CCNP, aflibercept, an anti‐VEGF therapy, was adopted as the positive control.^[^ ^26^ ^]^ As shown in Figure 6c,d and M@CCNP demonstrated superior efficacy to aflibercept in reducing leakage, with a reduction rate of 68.4% and 54.2% in the leakage area, respectively. Concerning neovascularization suppression, M@CCNP and aflibercept showed comparable efficacy, with a reduction rate of 84.4% and 81.3% in the neovascularization area, respectively (Figure 6f,g). Besides, both M@CCNP and aflibercept effectively restored retinal structural integrity according to the OCT imaging (Figure 6e) and histological analysis (Figure 6i,j).

In summary, M@CCNP exhibited excellent efficacy in mitigating neovascularization, alleviating leakage, and restoring the integrity of the fundus structure with good biocompatibility and efficient fundus accumulation. M@CCNP displayed non‐inferior therapeutic efficacy to aflibercept, a currently established first‐line therapy, and surprisingly, significantly outperformed aflibercept in reducing leakage, suggesting M@CCNP may offer superior potential for preserving or improving vision in practical therapeutic applications.

### 2.6 M@CCNP Suppressed In Vivo Oxidative Stress and Inflammation

**Figure 7:**
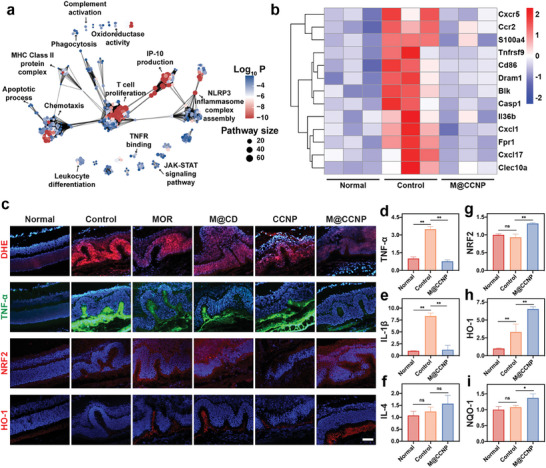
The transcriptome analysis of choroidal flat mounts of normal mice, CNV mice with intravitreal injection of 2 µL of saline and M@CCNP (MOR: 17.8 µM, CCNP: 1 mM), and the in vivo anti‐ROS, anti‐inflammatory, and NRF2 activation effects of M@CCNP. The enrichment network from GO enrichment analysis (a) and the heatmap (b) of differentially expressed genes. (c) Representative confocal fluorescent images of cryopreserved sections stained with DHE, TNF‐α, NRF2, and HO‐1; scale bar = 50 µm. (d‐i) Q‐PCR analysis of TNF‐α, IL‐1β, IL‐4, NRF2, HO‐1, and NQO‐1. Results in (d‐i) are presented as mean ± SD; n = 3; ns, *p* > 0.05; *p < 0.05; **p < 0.01.

The transcriptome profile of choroidal flat mounts collected from different groups offered a comprehensive overview of the underlying mechanisms of M@CCNP treatment. Principal component analysis (PCA) revealed significant differences among the normal, control (saline administration), and M@CCNP groups (Figure S34, Supporting Information). A comparison between the M@CCNP and control groups identified 1031 differentially expressed genes, comprising 228 upregulated and 803 downregulated genes in the M@CCNP group (Figure S35 and S36, Supporting Information). Similarly, a comparison between the control and normal groups revealed 1356 differentially expressed genes. Among these genes, 278 were upregulated in response to CNV modeling but downregulated following M@CCNP treatment, while 35 genes exhibited the opposite expression pattern (Figure S36, Supporting Information). Gene Ontology (GO) and Kyoto Encyclopedia of Genes and Genomes (KEGG) enrichment revealed enriched pathways associated with both innate and adaptive immune responses, particularly cytokine‐ and chemokine‐signaling pathways, phagocytosis, complement activation, apoptosis, and pyroptosis (**Figure** **7**a; Figures S37 and S38, Supporting Information). Additionally, M@CCNP treatment was implicated in the regulation of oxidoreductase activity, contributing to its ROS scavenging capabilities. To further specify the differential expression genes, heatmap was utilized to illustrate the hierarchical clustering analysis results, revealing that M@CCNP significantly down‐regulated genes associated with inflammation and ROS, including chemokines (Cxcr5, Ccr2, Cxcl1, Cxcl17), cytokines (Tnfrsf9, IL36b), G‐protein coupled receptor with potential inflammation and ROS regulation effects (Fpr1),^[^ ^27^ ^]^ and M1 subset markers (CD86).^[^ ^28^ ^]^ Other differentially expressed genes with specific functions, such as cell migration and invasion (S100a4),^[^ ^29^ ^]^ autophagy regulation (Dram1),^[^ ^30^ ^]^ and apoptosis regulation (Blk, Caspase 1),^[^ ^31^ ^]^ may also contribute to its therapeutic efficacy (Figure 7b). In summary, the transcriptomic analysis confirmed M@CCNP's synergistic targeting of both oxidative stress and inflammation. Moreover, the M@CCNP treatment presented the potential of apoptosis and autophagy suppression, as well as inhibition of cell migration and invasion, which might contribute to its excellent in vivo anti‐angiogenesis effects.

To further validate the in vivo suppression effects of M@CCNP on oxidative stress and inflammation, immunofluorescence staining and Q‐PCR were conducted. As illustrated in Figure 7c, compared with the control group, the M@CCNP group showed significantly reduced levels of oxidative stress and inflammation, as indicated by dihydroethidium (DHE) and TNF‐α staining, respectively. The inhibition of oxidative stress and inflammation was also seen in the MOR, M@CD, and CCNP groups, but not as effective as in the M@CCNP group. Q‐PCR analysis demonstrated that M@CCNP markedly reduced pro‐inflammatory cytokines TNF‐α and IL‐1β, while anti‐inflammatory cytokine IL‐4 showed no significant difference (Figure 7d–f), which was in accordance with the in vitro experiments.

Furthermore, we assessed the regulation effects of M@CCNP on the NRF2 signaling pathway in vivo. As illustrated in Figure 7c, the MOR‐containing groups exhibited significant upregulation of NRF2 and HO‐1 expression, whereas the change in the CCNP group was less pronounced, as observed at the cellular level. The in vivo activation of the NRF2 signaling pathway was further verified by Q‐PCR analysis (Figure 7g–i).

## 3 Discussion and Conclusion

AMD presents significant challenges to the growing aging population worldwide. With its rising prevalence, severe vision impairment, and the limited efficacy of current treatments, there is an urgent need for novel therapeutic approaches that offer higher response rates and improved outcomes. The two‐level strike model provides a more comprehensive understanding of AMD, suggesting that early‐stage interventions targeting upstream processes may be more effective than the traditional focus on downstream VEGF signaling.

In line with this model, we developed a biocompatible and easily fabricated supramolecular strategy that combines the anti‐inflammatory effects of MOR with the regenerative ROS‐scavenging properties of CCNP. α‐CD served as a key molecular bridge, coating the nanoceria surface via O─Ce bonds and encapsulating MOR through host–guest interactions. Possessing the typical fluorite cubic structure and high Ce^3+^/Ce^4+^ ratio, M@CCNP exhibited robust self‐regenerating antioxidant capabilities and concentration‐dependent enzyme‐mimicking activity.

In an in vitro LPS‐stimulated macrophages model, M@CCNP could be efficiently taken up by macrophages through active endocytosis, and demonstrated strong anti‐oxidative and anti‐inflammatory effects. The ROS scavenging properties were primarily attributed to CCNP, while MOR contributed to significant anti‐inflammatory activity and potent NRF2 activation.

M@CCNP demonstrated superiority in AMD treatment, reaching 84.4% in inhibiting neovascularization area and 68.4% in alleviating leakage area, showing non‐inferior efficacy to current first‐line anti‐VEGF therapy in neovascularization alleviation and even superior in leakage suppression, which potentially leads to better vision outcome. This high efficacy can be attributed to several factors: (1) efficient fundus accumulation, which is likely attributable to the small size and nearly neutral charge of M@CCNP;^[^ ^1^, ^32^ ^]^ (2) potent in vivo ROS scavenging, which alleviated oxidative stress and prevented further molecular damage; (3) activation of the NRF2 signaling pathway, which downregulated pro‐inflammatory M1 macrophages and their associated cytokines without increasing VEGF‐secreting M2 macrophages, thus preventing unwanted vascular proliferation. Additionally, the distinct pharmacokinetic profiles of M@CCNP align with the two‐level strike pathogenesis of AMD, which will be blocked in the initial first strike via the rapid alleviation of existing inflammation by MOR and sustained inhibition of oxidative stress by nanoceria. Moreover, M@CCNP demonstrated no significant toxicity in the fundus and major organs, exhibiting excellent local and systemic biocompatibility.

Meanwhile, several limitations in our current work require future exploration. First, laser‐induced mice CNV model, the most widely used animal model for wet AMD,^[^ ^33^ ^]^ was employed in our in vivo evaluation, but it has been noted for representing an acute, trauma‐induced neovascularization process.^[^ ^34^ ^]^ While transgenic models, such as Vldlr^−/−^ mouse model,^[^ ^35^ ^]^ may better mimic the chronic, progressive age‐related degeneration observed in clinical wet AMD, and contribute to further assessment of the long‐term therapeutic efficacy of M@CCNP. Second, the types of loading drugs were limited, as only α‐CD was employed in the delivery system, which could be addressed by exploiting other cyclodextrins for the supramolecular engineering of nanoceria. Third, our current results only provide preliminary insights into the biodistribution and retention of M@CCNP. Limited by the particularly tiny fundus tissue in the mouse model and detection limit of HPLC‐MS, fluorescein was used to mimic the biodistribution and retention of MOR, while it was less reliable for tracking CCNP distribution over extended periods owing to the release behavior, thus ICP‐MS was employed to measure the pharmacokinetic behavior of CCNP. In the future, we plan to obtain more comprehensive pharmacokinetic data using larger animal models, such as rabbits and pigs, to better evaluate the biodistribution and long‐term therapeutic effects of M@CCNP.

In summary, our M@CCNP provides a promising therapeutic option for comprehensive AMD mitigation via blocking the two‐level strike. Additionally, the supramolecular engineering strategy offers new insights into harnessing the medical potential of nanoceria as a drug delivery platform with intrinsic ROS elimination properties, particularly in oxidative stress‐related diseases.

## 4 Experimental Section

### Synthesis of the M@CCNP

CCNP was synthesized using a one‐pot hydrothermal method.^[^ ^14^ ^]^ Briefly, 1 mmol cerium nitrate hexahydrate and 1 mmol α‐CD were dissolved in 15 mL of deionized water under vigorous stirring at room temperature. Then, the mixture was added dropwise with 2 mL of 7.5 M NaOH solution and stirred vigorously with the color changing from white to brown. In an argon atmosphere, the brown mixture was then heated at 125 °C for 6 h in a Teflon‐lined stainless‐steel autoclave. Once cooled, the precipitates were separated by centrifugation at 5000 rpm for 10 min, then washed three times with deionized water and salt solution to remove ionic residues. The current reaction temperature and α‐CD/Ce molar ratios were determined according to the balancing of particle size and relative conversion rate of α‐CD (Figures S39 and 40, Supporting Information). The resulting CCNP was dispersed in deionized water, and the molality of cerium was calculated by ICP‐MS. The content of α‐CD in the nanoparticles was measured by anthrone spectrophotometric methods.^[^ ^18^ ^]^ Then, MOR was added to the CCNP solution with MOR/α‐CD = 1:1 stoichiometry.^[^ ^13^ ^]^ The actual loaded MOR was measured according to the standard curve of MOR using UV–vis spectrophotometer at 228 nm.

### Molecular Modeling

All calculations were carried out with CP2K package (version 7.1) in the framework of GFN‐xTB, based on the tight‐binding quantum chemical method.^[^ ^36^ ^]^ A plane‐wave density cutoff of 500 Ry was adopted. The long‐range *van der Waals* interaction is described by the DFT‐D3 approach. All the structures were fully relaxed by CP2K with BFGS scheme, and the force convergence criterion was set to 4.5 × 10^−4^ hartree/bhor.

### In Vitro Stability Evaluation of CCNP

The freshly synthesized CCNP was dissolved in PBS and stored at 4 °C. The hydrodynamic size of CCNP was tested at varied time points (at Day 0, 7, 14, 21, and 28 post‐synthesis) and before/after lyophilization.

### In Vitro MOR Release Behavior of M@CCNP

To assess the in vitro release behavior of MOR in M@CCNP, first, the characteristic absorption band of MOR at 228 nm in release medium (PBS with 0.5% (v/v) Tween‐80, pH 7.4) was determined by ultraviolet absorption spectrum, followed by plotting of the standard curve. Then, the release rate of MOR was measured using the dialysis method. 1 mL of M@CCNP containing 1 mg·mL^−1^ MOR was encapsulated in a dialysis bag with a cutoff molecular weight of 3500, immersed in 19 mL of release medium, and incubated in a thermostatic incubator at 37 °C with a shaking speed of 100 rpm. 500 µL of releasing medium was collected at different time points and measured by UV–vis spectroscopy at 228 nm, with an equal volume of fresh release medium added back to the releasing system to maintain a constant total volume.

### In Vitro Anti‐Oxidative Abilities

1) Raman spectroscopy experiment. Raman spectra were recorded of the sample solution containing: 10 mg M@CCNP; 10 mg M@CCNP + 0.1 mL of 10% H_2_O_2_ at the time intervals of 0 min and 60 min after the mixing under 488 nm laser excitation. 2) XPS experiment. Ce^3+^/Ce^4+^ patterns of the synthesized nanoparticles and the ability of spontaneous interconversion between Ce^3+^ and Ce^4+^ were determined by XPS under conditions of: 10 mg M@CCNP; 10 mg M@CCNP + 0.15 mmol H_2_O_2_ at the time intervals of Day 0 and Day 6. 3) ESR experiments. ESR experiments were conducted to test the free radicals scavenging ability of CCNP and M@CCNP.^[^ ^37^ ^]^ Samples were prepared with the following procedure: 100 µL of 8 mM/16 mM CCNP/M@CCNP samples was added to the mixture solution of 50 µL of 1 mM FeSO_4_ and 10 µL of 1 M DMPO, subsequently initiated the reaction by adding 50 µL of 10 mM H_2_O_2_. After 5 min, the reaction solutions were drawn into a capillary for the ESR test. 4) Multienzyme mimetic experiment. To evaluate the multienzyme mimetic activities of CCNP and M@CCNP, total SOD assay (Beyotime, S0101S), DPPH free radical scavenging capacity assay (Nanjing Jiancheng Bioengineering Institute, A153‐1‐1), HORAC assay (Nanjing Jiancheng Bioengineering Institute, A018‐1‐1), and CAT assay (Nanjing Jiancheng Bioengineering Institute, A007‐2‐1) were conducted according to the suggested protocols from the manufacturer. Besides, a kinetic study was performed to evaluate the oxidase‐mimic activity of CCNP according to previously reported protocols.^[^ ^38^ ^]^ Briefly, a mixture of TMB with varied concentrations (0.3–4.8 mM) and 0.375 mM CCNP in sodium acetate buffer (pH 5.5) was incubated at room temperature for 60 min, and the newly formed oxTMB was monitored by UV–vis spectra at 652 nm. The oxTMB concentration was quantified using Lambert‐Beer's law, and the reaction rates at various TMB concentrations were calculated based on the slopes of initial absorbance change with time. 5) The record of the color change experiment. Adding 1 mmol H_2_O_2_ into 10 mg CCNP/M@CCNP dissolved in deionized water, and after 6 days, 1 mmol H_2_O_2_ was added to the solutions again. Images were taken to record the color change of CCNP and M@CCNP.

### Cell Viability Assays

CCK‐8 (Beyotime, C0037) and Calcein/PI live‐dead assays (Beyotime, C2015S) were performed to evaluate the in vitro cytotoxicity. For CCK‐8 assays, RAW264.7 and HUVEC were seeded in 96‐well plates overnight at a density of 1 × 10^4^ cells per well, followed by incubation with M@CCNP of varied concentrations for 24 h. Then, 100 µL of fresh media containing 10 µL of CCK‐8 solutions were added to each well according to the manufacturer's protocol. The absorbances were then measured by a microplate reader at 450 nm. For the live‐dead assay, RAW264.7 and HUVEC cells were cultured in 12‐well plates overnight at a density of 5 × 10^5^ and 2 × 10^5^ cells per well, respectively. After 24 h of incubation with M@CCNP, the culture media was replaced by Calcein AM/Propidium Iodide solution and incubated for an additional 30 min, followed by being photographed using a fluorescence microscope.

### Cellular Uptake

RAW264.7 cells were seeded in 24‐well plates overnight at a density of 1 × 10^5^ per well for fluorescence imaging and in 6‐well plates overnight at a density of 8 × 10^5^ cells per well for flow cytometric analysis, followed by incubation of Fluorescein@CCNP or fluorescein (with equivalent fluorescein concentration) for 0, 1, 2, and 4 h. For the exploration of the cellular uptake mechanism, RAW264.7 cells were incubated with 10 µg·mL^−1^ chlorpromazine, 0.5 mM amiloride, and 0.2 mM genistein at 37 °C and fresh media at 4 °C, and then prepared for fluorescence imaging and flow cytometric analysis.

### In Vitro Anti‐Oxidative and Anti‐Inflammatory Effects of M@CCNP in Cells

RAW264.7 cells were seed overnight in 6‐well plates at a density of 8 × 10^5^ cells per well for flow cytometric analysis, and upon cell crawling slides in 24‐well plates at a density of 2 × 10^5^ cells per well for fluorescence imaging. 1 µg·mL^−1^ LPS was used for ROS and inflammation stimulation, and the dosage of M@CCNP was determined based on cell viability assays and the dose‐response curve of intracellular M1 inhibition and NRF2 stimulation (Figure S41, Supporting Information). Intracellular ROS was measured by DCFH‐DA assay (Beyotime, S0033S). The cells were treated with 1 µg·mL^−1^ LPS for 1 h for ROS stimulation, followed by the addition of MOR, M@CD, CCNP, and M@CCNP (MOR: 3.55 µM, CCNP: 200 µM) and incubation for 24 h. After the incubation, cells were rinsed and treated with DCFH‐DA solution for 30 min and then prepared for fluorescence imaging and flow cytometric analysis. The anti‐inflammatory effects of M@CCNP were determined by fluorescence imaging and flow cytometric analysis of M1/M2 subsets markers, and Q‐PCR analysis of inflammatory cytokines. The cells were treated with 1 µg·mL^−1^ LPS for 1 h for inflammation stimulation, followed by the addition of MOR, M@CD, CCNP, and M@CCNP (MOR: 3.55 µM, CCNP: 200 µM) and incubation for 4 h. For fluorescence imaging, TNF‐α antibody was used to detect the M1 subset of macrophages, TGF‐β antibody was used to detect the M2 subset of macrophages, and NRF2. For flow cytometric analysis of M1/M2 subsets, iNOS and CD206 served as M1 and M2 markers, respectively. The treated cells were incubated with PE‐conjugated Nos2 (iNOS) antibody and APC‐conjugated CD206 antibody, and then prepared for flow cytometric analysis. The impact of M@CCNP on the NRF2 signaling pathway was evaluated by: 1) fluorescence imaging analysis of cellular localization and fluorescence intensity of NRF2, which was calculated by ImageJ software; 2) Q‐PCR analysis and 3) Western blotting analysis of key protein of the NRF2 signaling pathway, including NRF2, HO‐1, and NQO‐1.

### Laser‐Induced CNV Mouse Model and Treatment Procedure

Laser‐induced CNV mouse model was induced following the standard protocol with slight modification.^[^ ^39^ ^]^ C57BL/6 mice (6‐8 weeks, female, 18 ± 2 g) were purchased from SLAC Laboratory, and housed in a controlled environment with constant temperature (22 ± 1 °C) and a regular light/dark (12 h/12 h) cycle with ad libitum access to food and water. The mice were first anesthetized by intraperitoneal injection with 60 mg·kg^−1^ pentobarbital and the pupils were dilated by 0.5% tropicamide eye drops. Four laser spots were induced surrounding the optic nerve in each of the four quadrants using krypton laser photocoagulation, air bubble sign was observed to confirm the successful rupture of the Bruch's membrane in each eye. Parameters were set as 532 nm wavelength, 50 µm spot size, 250 mW power, and 100 ms duration. After 5 days after the photocoagulation, the fundus images, FFA images, and OCT images were captured. Four laser spots with hyperfluorescent leakages indicated successful CNV lesion induction, while those with fundus hemorrhage following laser application were excluded from further treatment. Specifically, FFA images were captured 300 s after intraperitoneal injection of 0.2 mL of 2% fluorescein sodium. Then, intravitreal injections were performed and CNV mice were randomly allocated into 5 groups with different administration. Briefly, CNV mice were anesthetized by intraperitoneal injection of 60 mg·kg^−1^ pentobarbital, followed by pupil dilation by 0.5% tropicamide eye drops and locally anesthetized by 0.5% proparacaine eye drops. A 30‐gauge needle was used to create a hole under the limbus, after which a cotton swab was used to gently massage the eye, removing some of the vitreous to prevent reflux of the drug solution or vitreous post‐injection. Following this, 2 µL of MOR, M@CD, CCNP, or M@CCNP (MOR: 17.8 µM, CCNP: 1 mM) in saline was injected into the vitreous through the original hole using a 33‐gauge Hamilton syringe. Saline‐only injections served as controls and 40 mg·mL^−1^ aflibercept (Eylea) served as the therapeutic benchmark. 0.3% levofloxacin ophthalmic gels were applied to the treated eyes to prevent infection. On Day 10, the fundus images, FFA images, and OCT images of CNV mice were captured again for comparison with the previous images, then the mice were sacrificed through overdose anesthesia, and eyeballs were collected for further analysis, including immunostaining of the choroidal flat mounts, H&E staining of the cross‐section of eyeballs, and RNA sequencing. All experiments were carried out and animals were maintained in accordance with the Association for Research in Vision and Ophthalmology (ARVO) statements for the use of animals in ophthalmic and vision research with ethics approval from the Ethics Committee of the Second Affiliated Hospital, School of Medicine, Zhejiang University (approval number: 2022–192).

### In Vivo Biodistribution of M@CCNP

To assess the pharmacokinetics of MOR, normal healthy mice received intravitreal injections of 2 µL of Fluorescein@CCNP in saline, and were sacrificed and harvested the eyeball at 0, 3, 6, 12, 24, and 48 h post‐administration. Cryopreserved sections were then made and observed. To evaluate the pharmacokinetics of nanoceria, normal healthy mice received intravitreal injections of 2 µL of 1 mM M@CCNP in saline, and were sacrificed and harvested the eyeball at 0, 3, 6, 24, 96, and 168 h post‐administration. The fundus tissue was collected and the cerium concentration of the samples was determined by ICP‐MS.

### In Vivo Anti‐Inflammatory Effects of M@CCNP

The in vivo anti‐inflammatory effects of M@CCNP were evaluated by immunostaining of TNF‐α and Q‐PCR analysis of TNF‐α, IL‐1β and IL‐4. The in vivo impact of M@CCNP on the NRF2 signaling pathway was evaluated by immunostaining of NRF2 and HO‐1.

### In Vivo Anti‐Oxidative Effects of M@CCNP

The in vivo anti‐oxidative effects of M@CCNP were evaluated by the DHE Assay Kit (Beyotime, S0063). Cryopreserved sections were incubated with 10 µM DHE in the dark for 30 min and washed three times, followed by being covered with DAPI Fluoromount‐G and observed under a fluorescence microscope.

### Safety Evaluation

Normal healthy mice without CNV modeling received intravitreal injections of 2 µL of 1 mM M@CCNP in saline at Day 0, and were sacrificed at Day 5 and Day 28 by overdose anesthesia, while CNV mice were sacrificed at Day 10. Blood samples were collected from the orbit for hematology and blood chemistry analysis. Major organs, including the liver, lung, heart, kidney, and spleen, were harvested and stained with H&E. Intraocular pressure was measured by a tonometer. Scotopic ERG was elicited at an intensity of 0.3 cd·s·m^−2^.

### Statistical Analyses

The results were presented as the mean ± standard deviation. Statistical analysis was performed by the GraphPad Prism software. For the comparison of two groups, an independent‐sample t‐test was conducted, while for the comparison of more than two groups, comparative studies of means were carried out in accordance with one‐way analysis of variance (ANOVA). The threshold with *P* < 0.05 was determined as statistical significance, and the difference was presented as follows: ns for no significant difference, * for *p* < 0.05, and ** for *p* < 0.01.

## Conflict of Interest

The authors declare no conflict of interest.

## Supporting information



Supporting Information

## Data Availability

The data that support the findings of this study are available from the corresponding author upon reasonable request.
