# Supplementary material for: Supramolecular Engineering of Nanoceria for Management and Amelioration of Age‐Related Macular Degeneration via the Two‐Level Blocking of Oxidative Stress and Inflammation
Source: Adv Sci (Weinh). 2025 Jan 10;12(9):2408436. doi: 10.1002/advs.202408436 (PMC11884525; doi:10.1002/advs.202408436)
Supplement: Supplementary file 1 — Supporting Information [file ADVS-12-2408436-s001.docx]

Supporting Information

**Supramolecular engineering of nanoceria for management and amelioration of age-related macular degeneration *via* the two-level blocking of oxidative stress and inflammation**

*Mingyu Xu^#^, Yifan Zhou^#^, Yufeng Xu^#^, An Shao, Haijie Han*, Juan Ye**

**Materials**

Moringin (MOR) was obtained from Yuanye Biotechnology. Cerium nitrate hexahydrate was purchased from Alfa Aesar. Fluorescein and α-cyclodextrin (α-CD) were provided by TCI. Anthrone and Tween-80 were purchased from Solarbio Life Sciences. DMPO, ferrous sulfate heptahydrate, and lipopolysaccharide (LPS) were purchased from Sigma-Aldrich. Chlorpromazine, amiloride, and genistein were obtained from APExBIO. Dulbecco’s modified Eagle’s medium (DMEM) was obtained from Corning. Penicillin-streptomycin was provided by Gibco, and fetal bovine serum (FBS) was purchased from AusGeneX. MilliQ Water 18.2 (MΩ cm^-1^) was obtained using a Millipore MilliQ Academic Water Purification System. Primers used were listed in **Table S**1, and antibodies were listed in **Table S**2.

**Characterizations**

The morphology of CCNP and M@CCNP was detected by TEM (HT7700), HRTEM (JEM-2100F), SAED (JEM-2100F), and X-ray diffraction (XRD, Bruker D2 Phaser). The hydrodynamic diameters and zeta potential of CCNP and M@CCNP were measured by dynamic light scattering (DLS, Malvern ZetaSizer Nano ZS90). The molality of cerium was measured by inductively coupled plasma mass spectrometry (ICP-MS, PerkinElmer NexION 2000). Ce 3d, O 1s, and C 1s spectra were surveyed by X-ray photoelectron spectroscopy (XPS, Thermo Scientific ESCALAB 250Xi). The free radicals scavenging ability of nanoparticles was tested on an electron spin resonance spectroscope (ESR, Bruker ESRA-300). To confirm the formation of MOR/α-CD inclusion complexes, ^1^H-NMR spectra (500MHZ, D_2_O: DMSO (v/v) =7:1, room temperature) (Bruker AVIII500M), 2D NOESY spectrum (600MHZ, D_2_O: DMSO (v/v) =7:1, room temperature) (Bruker AVIII500M), FTIR spectra (Thermo Scientific Nicolet iS20), and UV-vis spectra (Thermo Scientific Evolution 300) were recorded. Raman spectra were recorded on a laser Raman microscope (Horiba LabRAM HR Evolution). For cellular experiments, the fluorescent images were recorded by confocal laser scanning microscopy (CLSM, Zeiss LSM900) and fluorescence microscopy (LEICA DMi8). Flow cytometric analysis was conducted by flow cytometer (BD FACSCanto II). For animal experiments, the fundus images, FFA images, and OCT images were captured by a retinal imaging Microscope (Phoenix Micron IV). Embedded eyeballs were sectioned by a cryopreserved slider (LEICA CM1950). Tissue slices were observed by a stereomicroscope (LEICA s9i) and intraocular pressure was measured by tonometer (Icare TonoLab).

**Cell culture**

RAW264.7 and HUVEC cell lines from American Type Culture Collection (ATCC) were incubated in 25 cm^2^ cell culture flasks and were cultured in DMEM supplemented with 10% fetal bovine serum and 1% Penicillin-streptomycin at 37 °C in a humidified 5% CO_2_ atmosphere.

**RNA isolation, cDNA synthesis, and quantitative PCR (Q-PCR)**

RNA isolation was conducted by RNA Extraction Kit (GeneSand, RE715) under the suggested protocol and a spectrophotometer (Thermo Scientific NanoDrop 2000c) was used for RNA quantitation. The quantified samples were used for reaction mixture preparation by PrimeScript RT Master Mix (Takara, RR036A), followed by reverse transcription reaction under the condition of 37°C for 15 min and 85°C for 5 s. The reverse transcribed cDNA was used for Q-PCR reaction mixture preparation by TB Green® Premix Ex Taq^TM^ reagents (Takara, RR420A), and the Q-PCR analysis was conducted using a QuantStudio 5 Real-Time PCR System (Applied Biosystems) as following parameters: Holding Stage: 95 °C for 30 s; PCR Stage: 40 cycles of 95 °C for 3 s, 60 °C for 30 s. Melt Curve Stage: 95 °C for 15 s, 60 °C for 60 s, 95 °C for 15 s. Quantitative analysis was performed using the comparative (2^-ΔΔCT^) method and each assay was normalized by amplifying the housekeeping cDNA m-GAPDH. **Table S**1 lists tested genes and sequences of forward and reverse primers.

**RNA sequencing and analysis**

Choroidal flat mounts were prepared for RNA isolation using TRIzol reagent, and then sent to Cosmos Wisdom Biotechnology Co., Ltd. (Hangzhou, China) for cDNA library construction and sequencing. Raw data analysis was conducted using R 4.3.2. Differential expression of gene levels was evaluated through a cut-off at p < 0.05, and the padjust for multiple testing. Absolute values of Fold changes were equal to or greater than 1.5.

**Western blotting**

Cell samples were lysed by RIPA Lysis Buffer (Beyotime, P0013B) containing 1mM phenylmethanesulfonyl fluoride (PMSF), protease inhibitor cocktail (Beyotime, P1008) on ice for 30 min with vigorous vortex for 15 s every 5 min, followed by being centrifuged at 1.4 × 10^4^ g for 15 min at 4 °C. The total protein concentration was measured by the BCA protein assay kit (Beyotime, P0010). Quantified protein was then mixed with loading buffer (Biosharp, BL502A), followed by being heated at 95°C for 5 min. Equal amounts of protein samples were individually loaded on 10% SDS-polyacrylamide gel (Vazyme, One-Step PAGE Gel Fast Preparation Kit) and after completing the electrophoresis, proteins in the gel were electronically transferred to the polyvinylidene difluoride (PVDF) membrane (Immobilon, IPVH00010). The PVDF membrane was blocked by QuickBlock™ Blocking Buffer for Western Blot (Beyotime, P0252) for 15 min and then incubated with primary antibodies overnight at 4 °C and secondary antibodies at room temperature for 1 h. The specific protein bands were visualized by a hypersensitive ECL Chemiluminescent Substrate (Biosharp, BL520a) and imaged by chemiluminescence imaging system (GE AI680RGB). The specific protein bands were further analyzed by ImageJ software.

**Immunofluorescence staining**

Eyeballs were quickly embedded in the OCT compound, and sectioned into 7 μm cryopreserved sections. The cryopreserved sections were fixed by 4% PFA for 20 min, permeabilized by 0.4% TritonX-100 for 20 min, and then blocked with 10% Goat Serum (Boster, AR1009) at room temperature for 60 min. The as-prepared sections were then incubated with the primary antibodies at 4 °C overnight and secondary antibodies at room temperature for 1 h. After being covered by DAPI Fluoromount-G^TM^, the slices were observed and captured by a fluorescence microscope. Fluorescence intensity and colocalization analysis of the captured images were quantified by ImageJ software.

**H&E staining**

The eyeballs of each group were collected, fixed in 4% PFA, and embedded in paraffin. The embedded eyeballs were sectioned into 4 μm vertical slices and subjected to H&E staining, followed by observation and recording using a stereomicroscope. The thickness of the lesions and surrounding normal spots were measured by ImageJ software.

**Table S**1. Primers used in quantitative RT-PCR

| Genes | Forward Primer (5' to 3') | Reverse Primer (5' to 3') |
| --- | --- | --- |
| m-GAPDH | ACCACAGTCCATGCCATCAC | CACCACCCTGTTGCTGTAGCC |
| m-NRF2 | TTCTTTCAGCAGCATCCTCTCCAC | ACAGCCTTCAATAGTCCCGTCCAG |
| m-HO-1 | CAAGCCGAGAATGCTGAGTTCATG | GCAAGGGATGATTTCCTGCCAG |
| m-NQO-1 | GCTGCAGACCTGGTGATATT | ACTCTCTCAAACCAGCCTTT |
| m-IL-4 | GGTCTCAACCCCCAGCTAGT | GCCGATGATCTCTCTCAAGTGAT |
| m-TNF-α | TAGCCCACGTCGTAGCAAAC | ACCCTGAGCCATAATCCCCT |
| m-IL-1β | TGCCACCTTTTGACAGTGATG | AAGGTCCACGGGAAAGACAC |
| m-iNOS | GGCATCGAAAAGCCCGAAAG | GGTCATCTTGTATTGTTGGGCTGAG |

**Table S**2. Antibodies used in Western blotting, flow cytometry, and immunostaining

| **Antibody** | **Company** | **Category number** | **Application** | **Dilution** |
| --- | --- | --- | --- | --- |
| Anti-CD206 Antibody | ThermoFisher | 17-2061-82 | Flow Cytometry | 1:200 |
| Anti-Nos2 (iNOS) Antibody | Biolegend | 696805 | Flow Cytometry | 1:200 |
| Anti-TNF-α Antibody | Abcam | ab1793 | Immunofluorescence | 1:200 |
| Anti-TGF-β Antibody | Abcam | ab66043 | Immunofluorescence | 1:400 |
| Isolectin-B4 | Invitrogen | I21413 | Immunofluorescence | 1:200 |
| Anti-NQO1 Antibody | Abcam | ab80588 | Western Blotting | 1:10000 |
| Anti-Heme Oxygenase 1 Antibody | Abcam | ab68477 | Western Blotting | 1:10000 |
| Anti-NRF2 Antibody | Cell Signaling Technology | 12721 | Western Blotting | 1:1000 |
| Anti-β-tublin Antibody | Proteintech | 10094-1-AP | Western Blotting | 1:10000 |
| Anti-rabbit IgG HRP-linked Antibody | Cell Signaling Technology | 7074S | Western Blotting | 1:1000 |
| Anti-mouse IgG HRP-linked Antibody | Cell Signaling Technology | 7076S | Western Blotting | 1:1000 |
| 594-conjugated Anti-rabbit IgG | Proteintech | SA00013-4 | Immunofluorescence | 1:500 |
| 488-conjugated Anti-mouse IgG | Proteintech | SA00014-3 | Immunofluorescence | 1:500 |

**
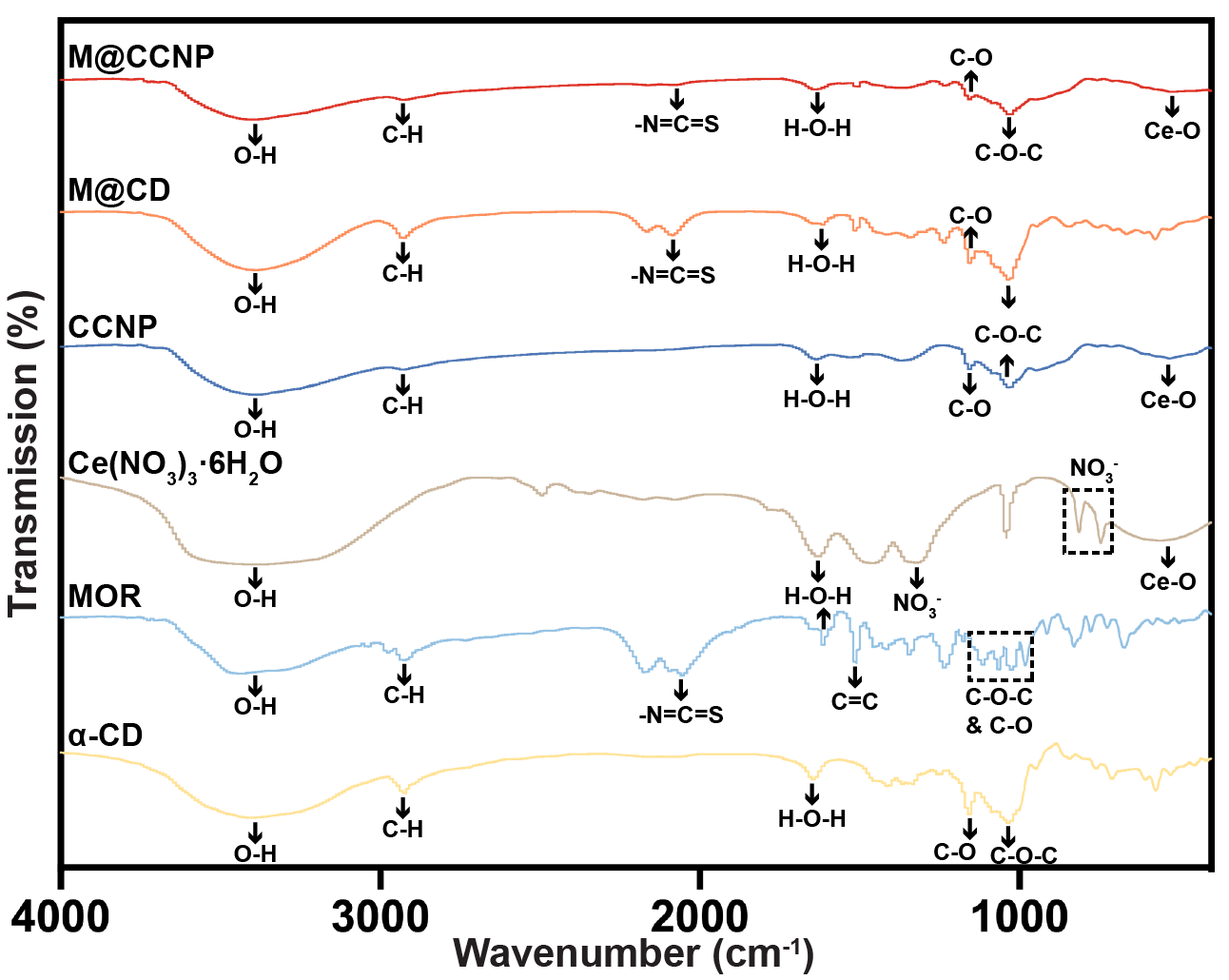
**

**Figure S**1**.** FTIR spectra of α-CD, MOR, Ce(NO_3_)_3_·6H_2_O, CCNP, M@CD, and M@CCNP.


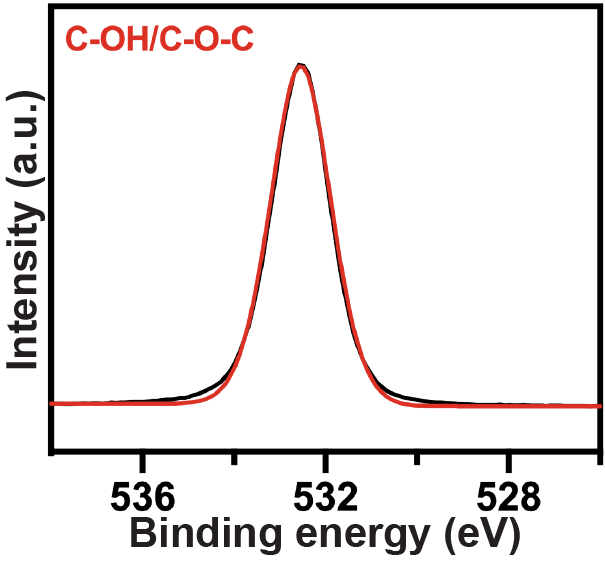


**Figure S**2**.** XPS analysis of O 1s of α-CD.


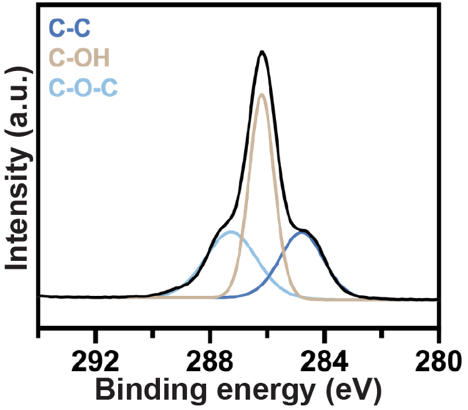


**Figure S**3**.** XPS analysis of C 1s of α-CD.

**
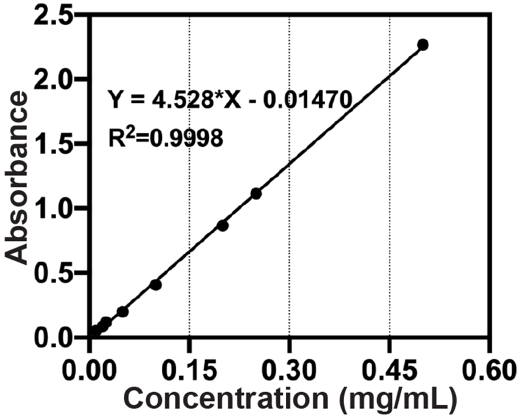
**

**Figure S**4**.** Standard curve of α-CD determined by anthrone spectrophotometric methods and quantified by UV-vis absorbance at the wavelength of 620 nm.

**
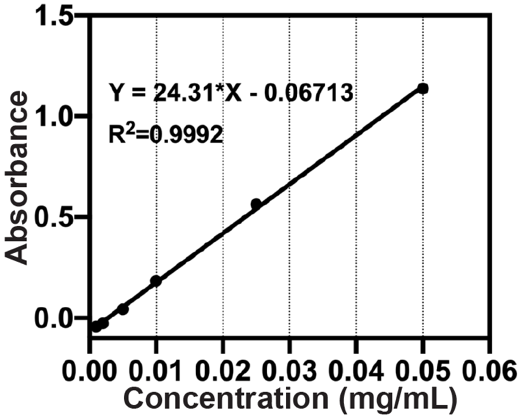
**

**Figure S**5**.** Standard curve of MOR dissolved in release medium (PBS containing 0.5% (v/v) Tween-80, pH 7.4).

**
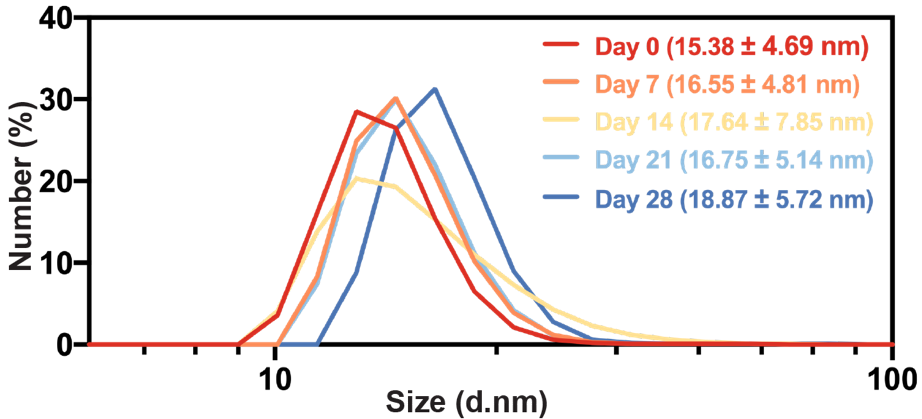
**

**Figure S**6**.** The hydrodynamic size of CCNP at varied time points. Results are presented as mean ± SD.

**
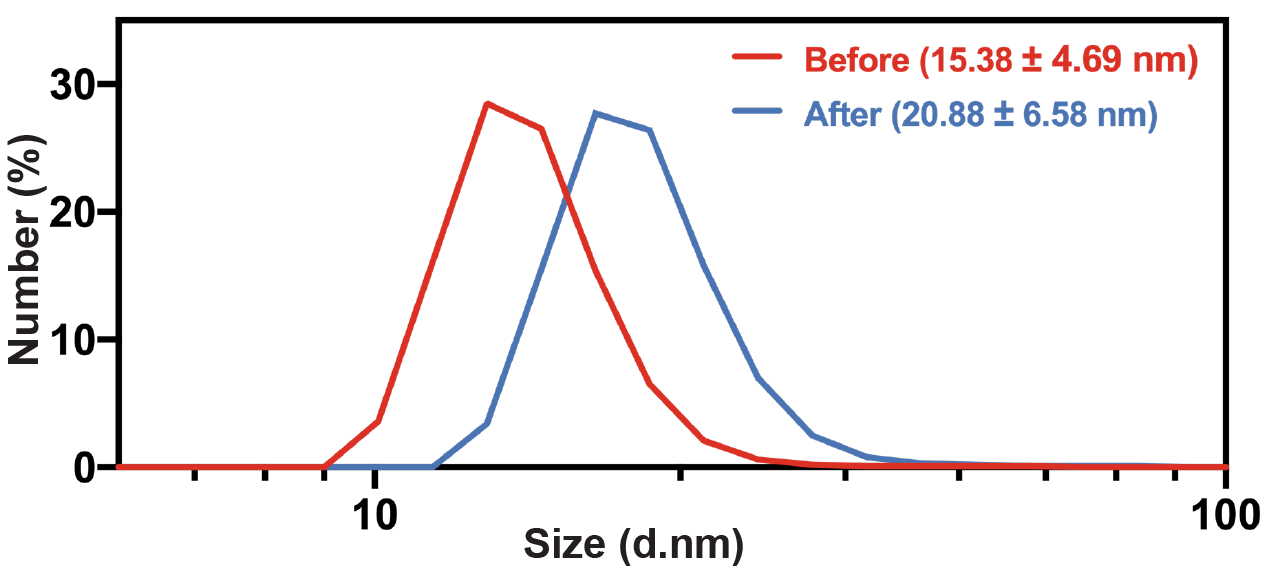
**

**Figure S**7**.** The hydrodynamic size of CCNP before/after lyophilization. Results are presented as mean ± SD.

**
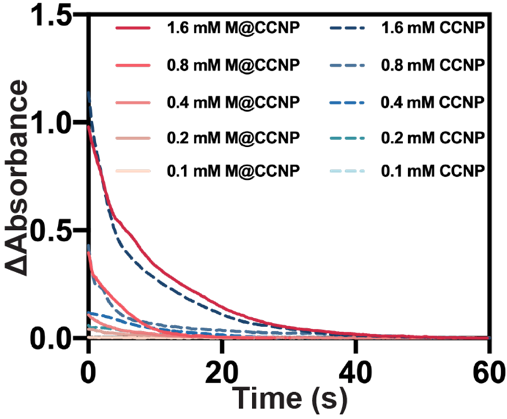
**

**Figure S**8. The UV-Vis absorbance change curve of CCNP and M@CCNP of different concentrations reacted with the substrate solution using the CAT assay at the duration of 60 seconds.

**
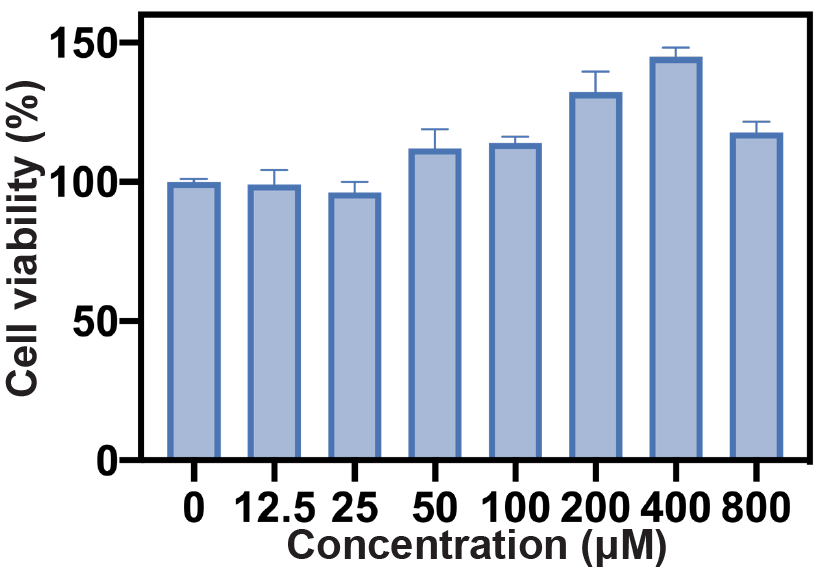
**

**Figure S**9**.** The cell viability of RAW264.7 after the 24 h treatment of different concentrations of M@CCNP using CCK-8 assay. Results are presented as mean ± SD, n = 3.


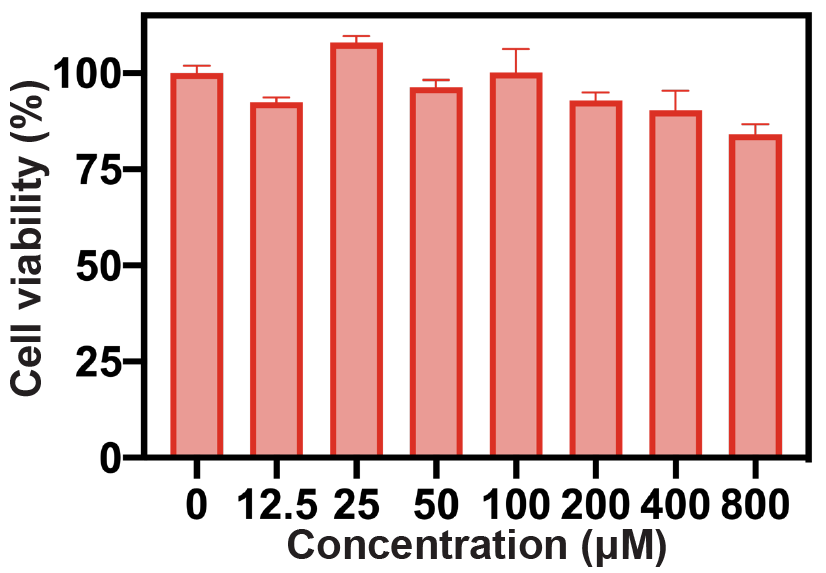


**Figure S**10**.** The cell viability of HUVEC after the 24 h treatment of different concentrations of M@CCNP using CCK-8 assay. Results are presented as mean ± SD, n = 3.

**
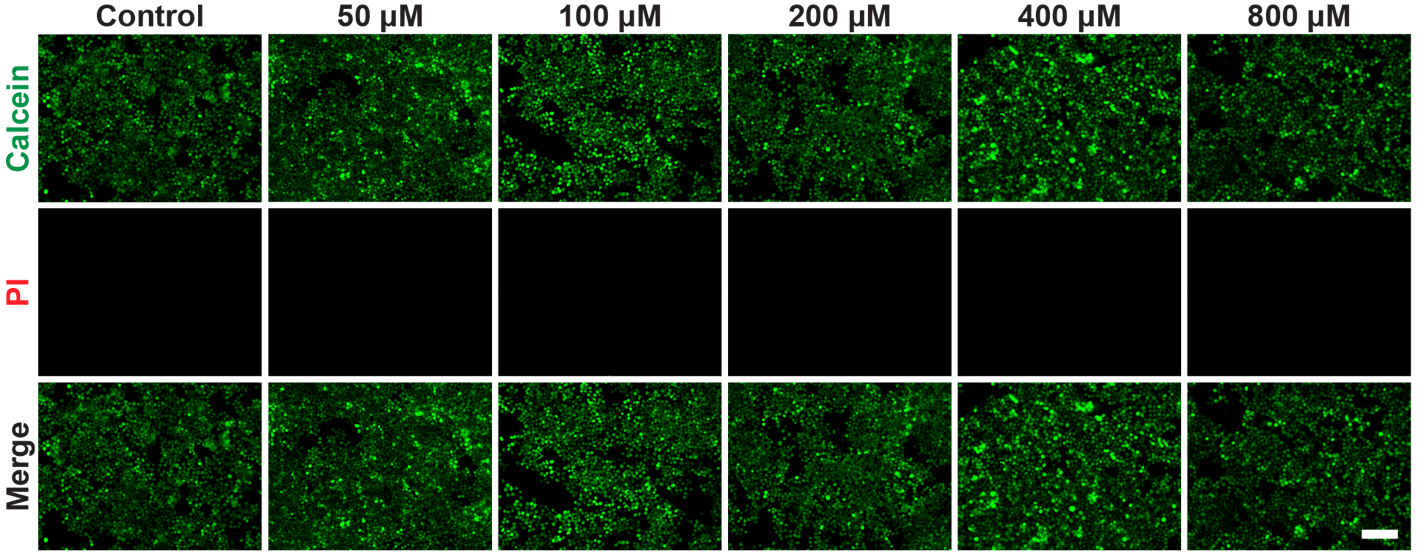
**

**Figure S**11**.** Representative live-dead images of RAW264.7 after 24 h treatment of different concentrations of M@CCNP; scale bar = 100 μm.


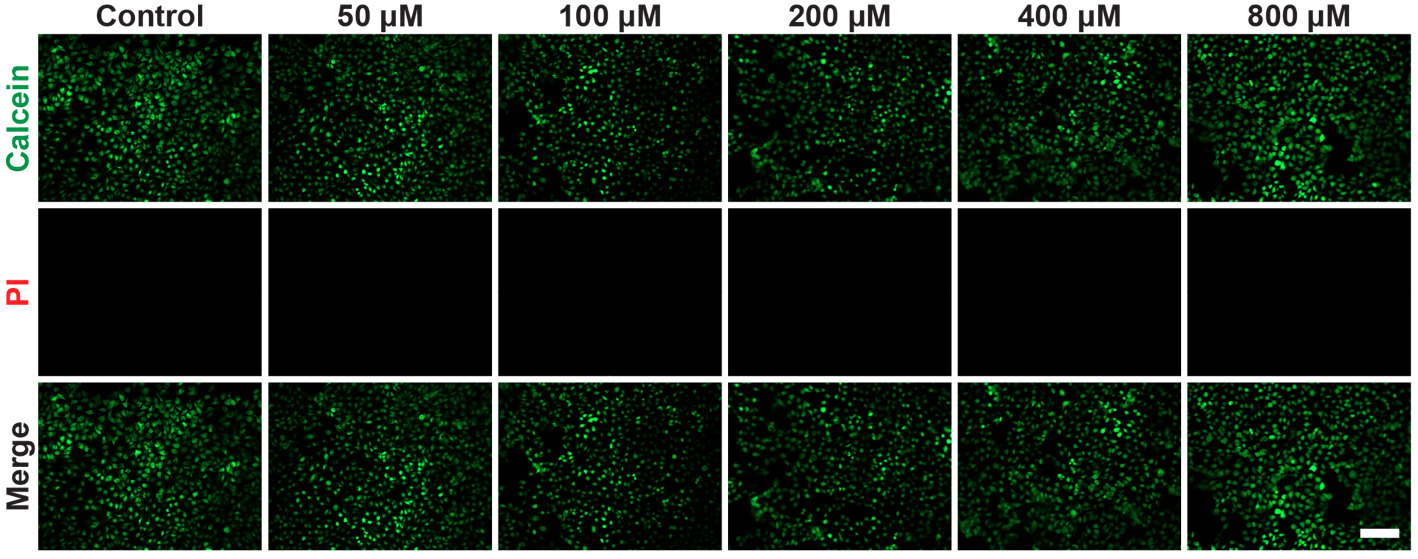


**Figure S**12**.** Representative live-dead images of HUVEC after 24 h treatment of different concentrations of M@CCNP; scale bar = 100 μm.

**
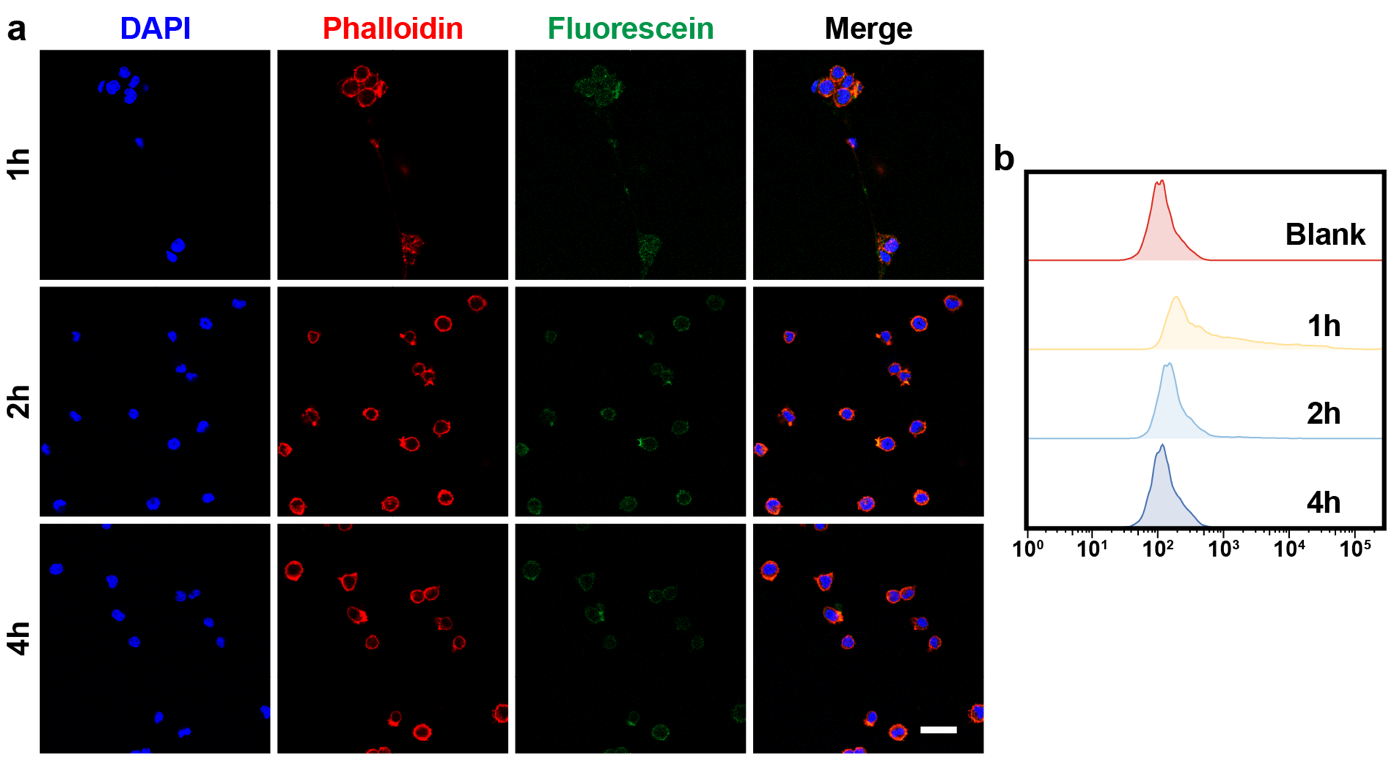
**

**Figure S**13**.** Representative fluorescence images (a) and the flow cytometric curve (b) of time-dependent cellular uptake behaviors of fluorescein at time points of 0, 1, 2, and 4 h; Green, Fluorescein; red, Phalloidin; blue, DAPI; scale bar = 25 μm.

**
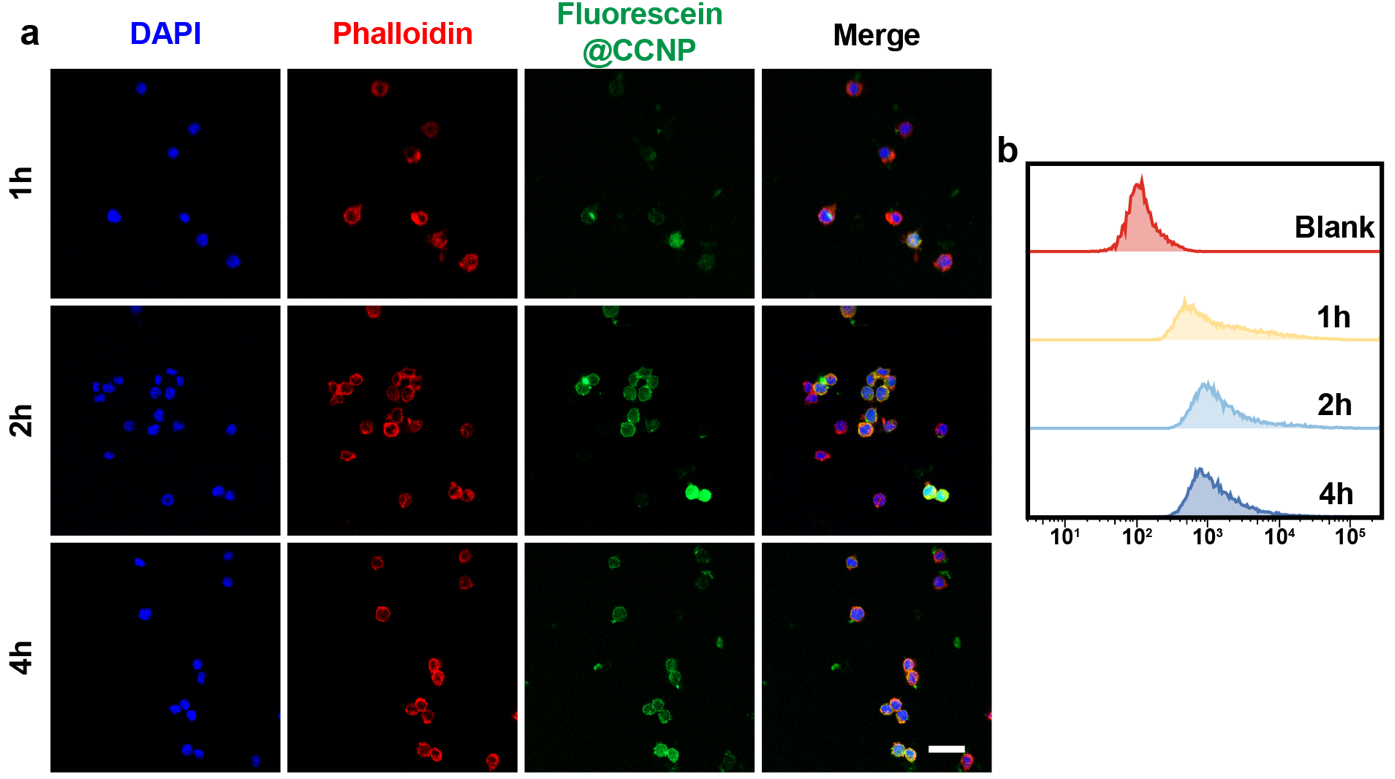
**

**Figure S**14**.** Representative fluorescence images (a) and the flow cytometric curve (b) of time-dependent cellular uptake behaviors of Fluorescein@CCNP at time points of 0, 1, 2, and 4 h; Green, Fluorescein@CCNP; red, Phalloidin; blue, DAPI; scale bar = 25 μm.

**
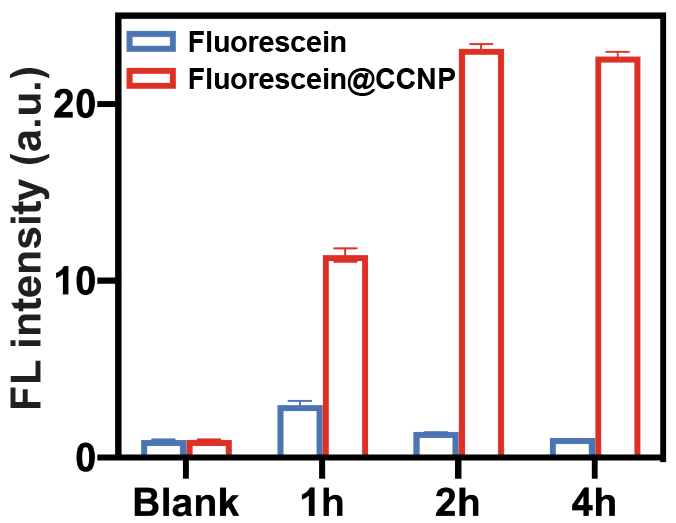
**

**Figure S**15**.** The quantitative analysis of fluorescent intensity according to the flow cytometry of fluorescein and Fluorescein@CCNP. Results are presented as mean ± SD, n = 3.


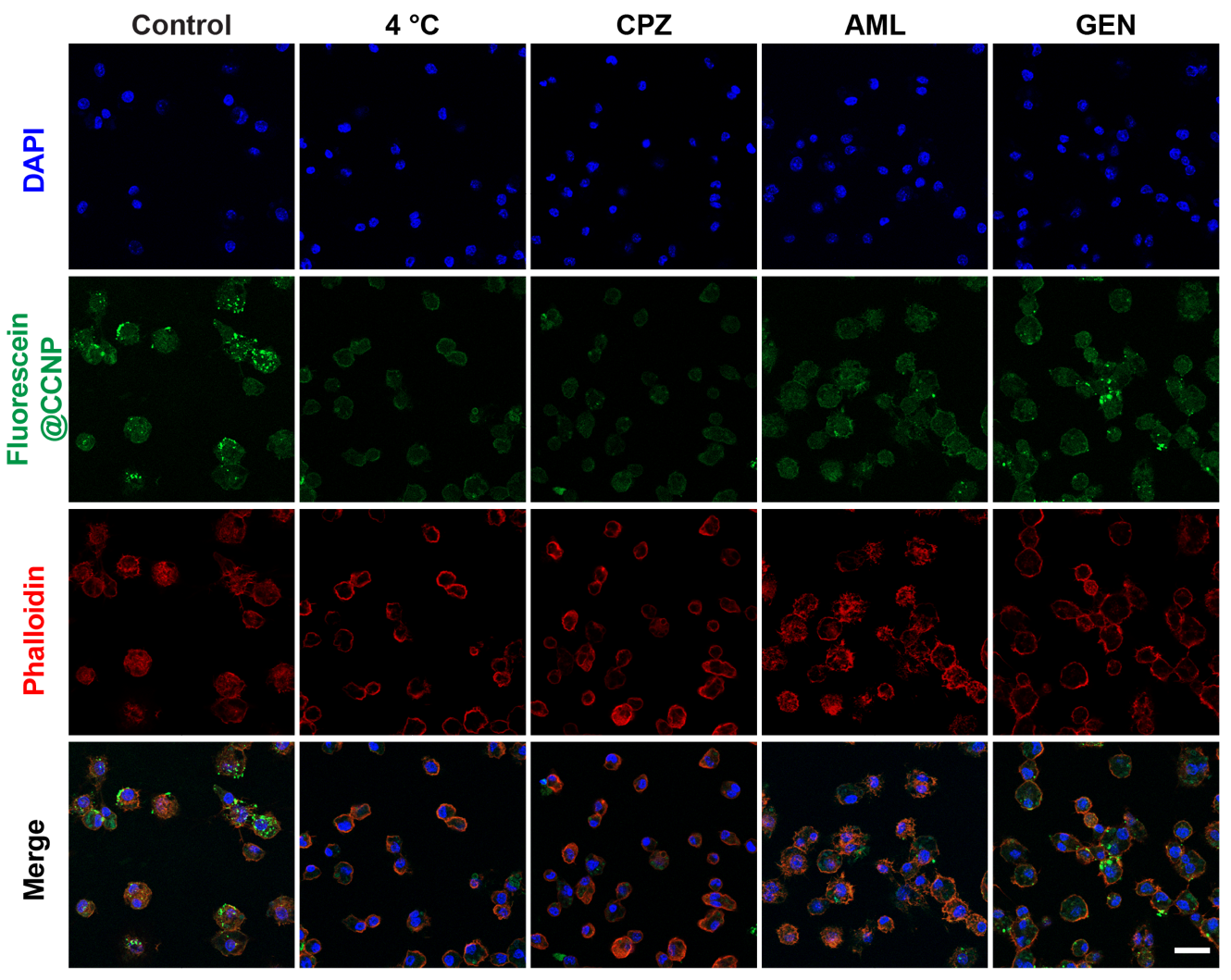


**Figure S**16. Representative fluorescent images reflecting the cellular uptake behaviors of Fluorescein@CCNP with varied treatments. Green, Fluorescein@CCNP; red, Phalloidin; blue, DAPI; scale bar = 25 μm.


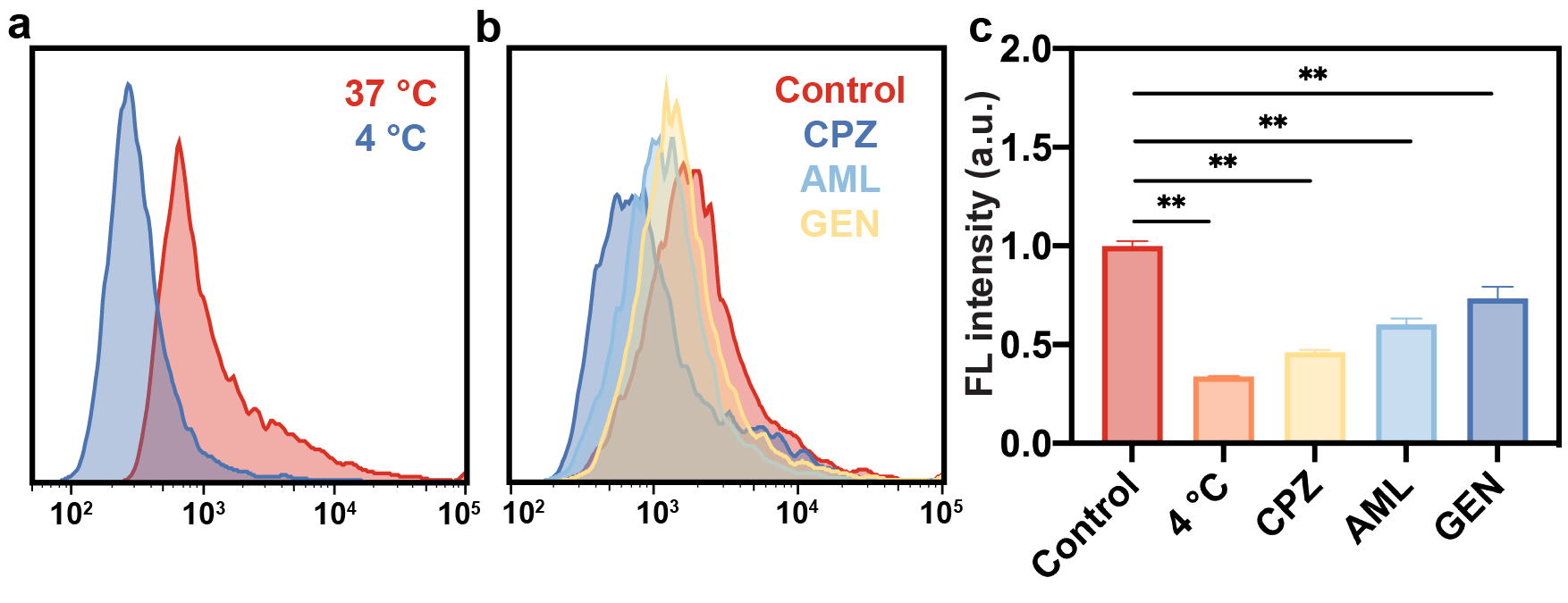


**Figure S**17. (a-b) Flow cytometric curves reflecting the cellular uptake behaviors of Fluorescein@CCNP with varied treatments, and (c) the corresponding quantitative results recorded by flow cytometry. Results in (c) are presented as mean ± SD; n = 3; ns, p > 0.05; *p < 0.05; **p < 0.01.


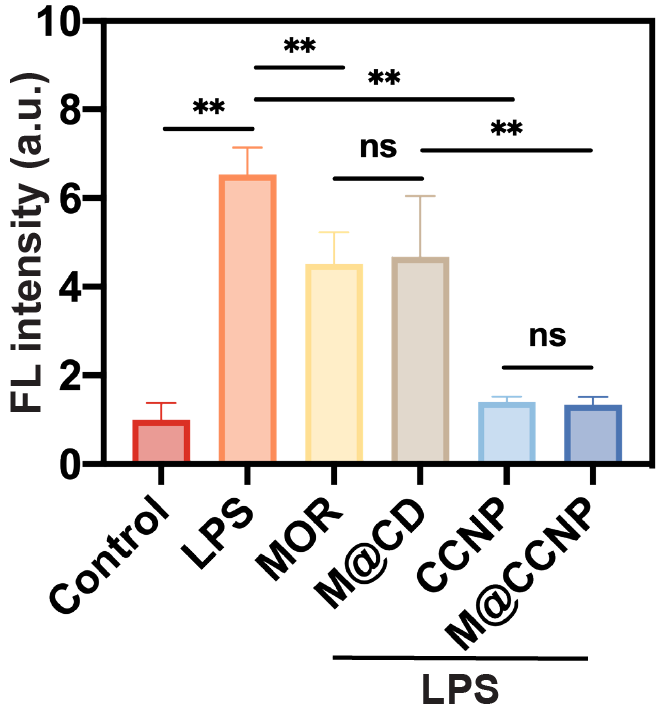


**Figure S**18**.** The quantitative results of fluorescent images reflecting intracellular ROS level of RAW264.7 with different treatments using DCFH-DA staining. Results are presented as mean ± SD; n = 3; ns, p > 0.05; *p < 0.05; **p < 0.01.

**
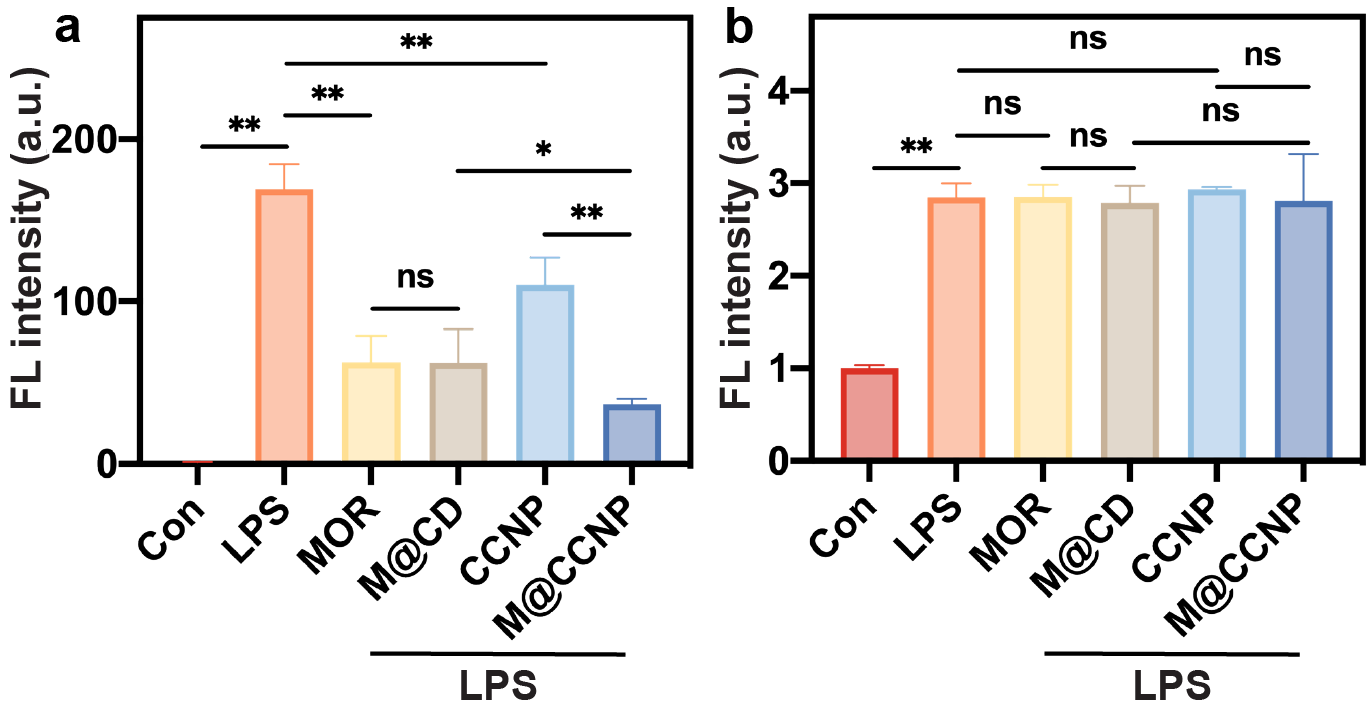
**

**Figure S**19**.** The quantitative analysis of PE-conjugated-iNOS (a) and APC-conjugated-CD206 (b) fluorescent signals detected by flow cytometry. Results are presented as mean ± SD; n = 3; ns, p > 0.05; *p < 0.05; **p < 0.01.


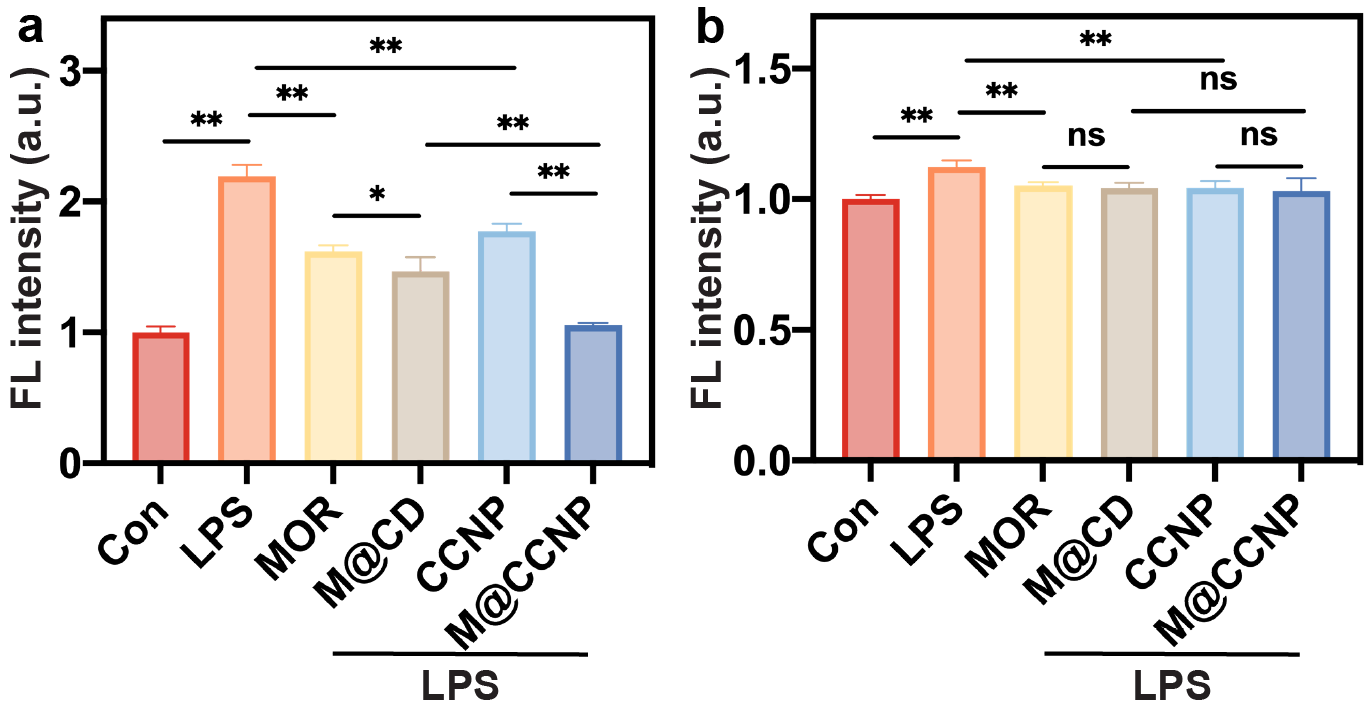


**Figure S**20**.** The quantitative analysis of RAW264.7 intracellular TNF-α (a) and TGF-β (b) expression with varied treatments according to the fluorescent images. Results are presented as mean ± SD; n = 3; ns, p > 0.05; *p < 0.05; **p < 0.01.

**
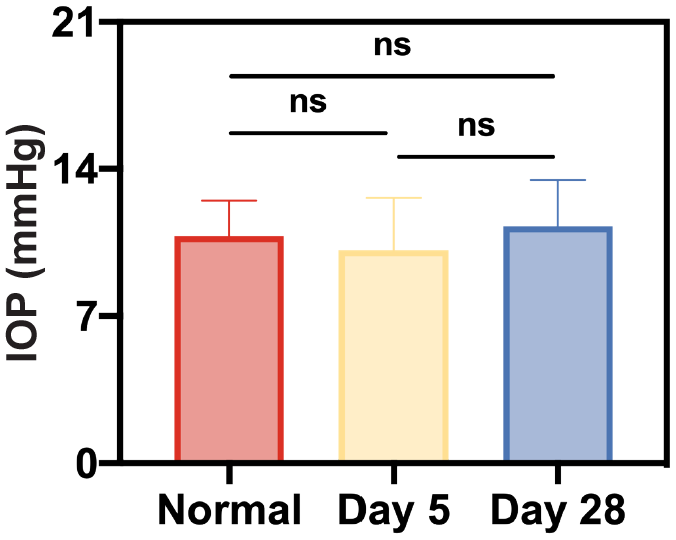
**

**Figure S**21**.** The intraocular pressure of normal mice, mice at 5 days and 28 days after intravitreal injections of 2 μL of M@CCNP (MOR: 17.8 μM, CCNP: 1 mM). Results are presented as mean ± SD; n ≥ 5; ns, p > 0.05; *p < 0.05; **p < 0.01.


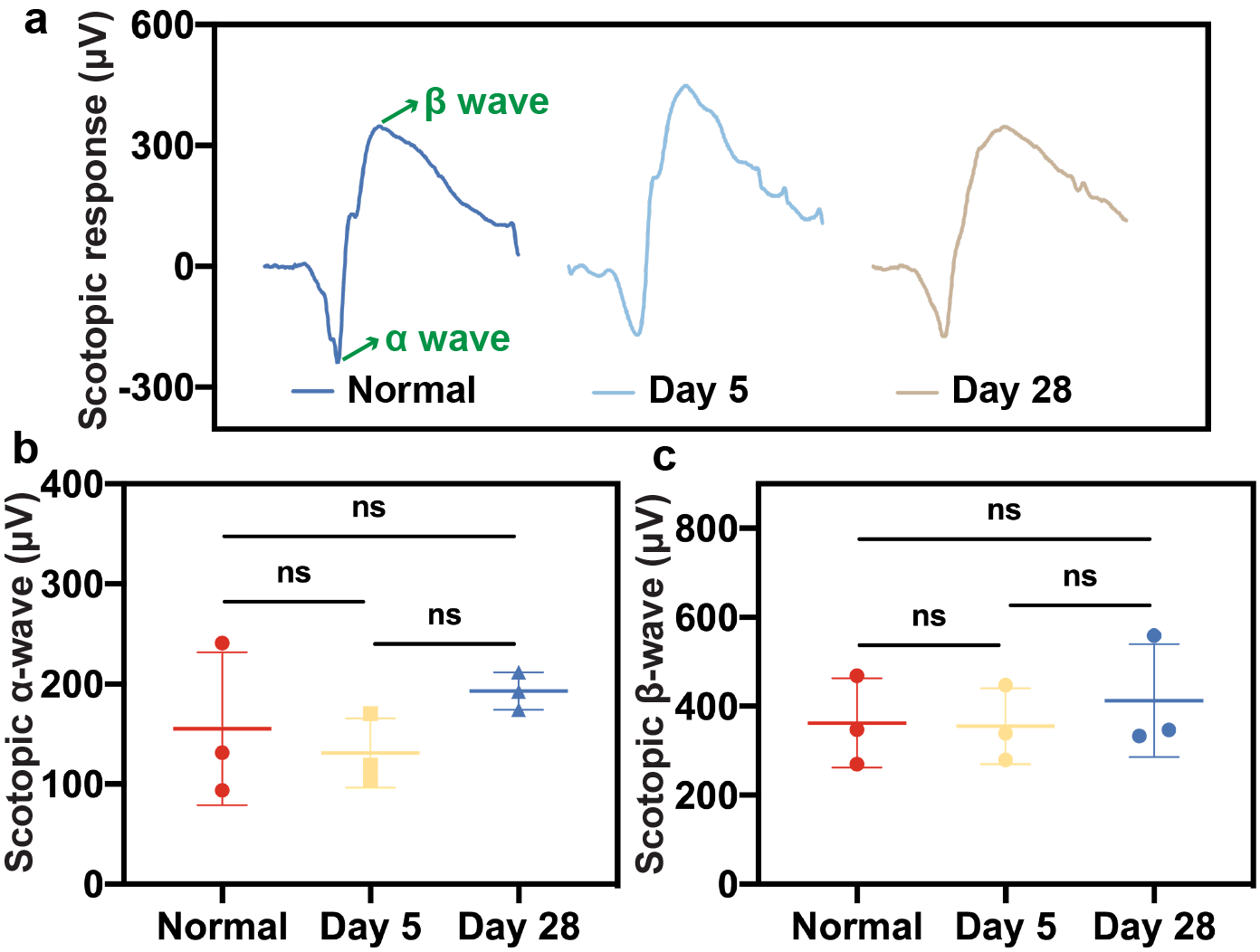


**Figure S**22**.** (a) The ERG spectra upon the scotopic response of normal mice, mice at 5 days and 28 days after intravitreal injections of 2 μL of M@CCNP (MOR: 17.8 μM, CCNP: 1 mM), and the corresponding quantitative results of the amplitude of α waves (b) and β waves (c). Results in (b, c) are presented as mean ± SD; n = 3; ns, p > 0.05; *p < 0.05; **p < 0.01.


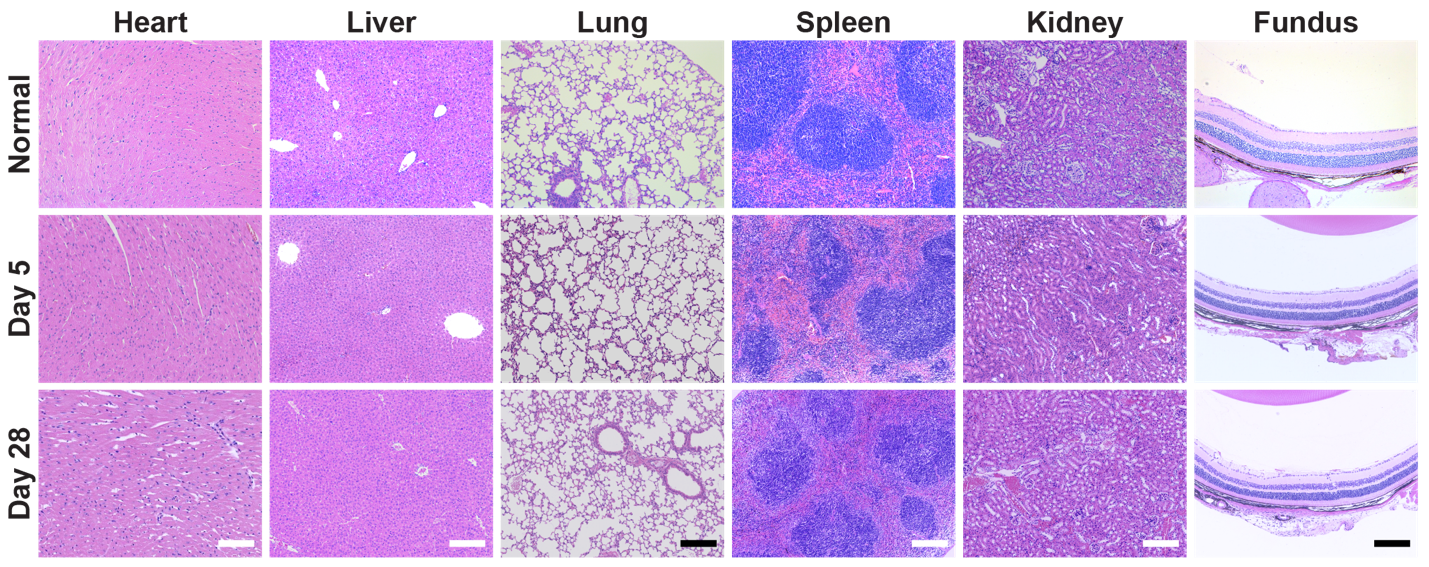


**Figure S**23**.** H&E staining of tissue sections of heart, liver, lung, spleen, kidney, and eye samples collected at normal mice, mice at 5 days and 28 days after intravitreal injections of 2 μL of M@CCNP (MOR: 17.8 μM, CCNP: 1 mM); scale bar = 100 μm (heart)/200 μm (liver, lung, spleen, kidney, and eye).


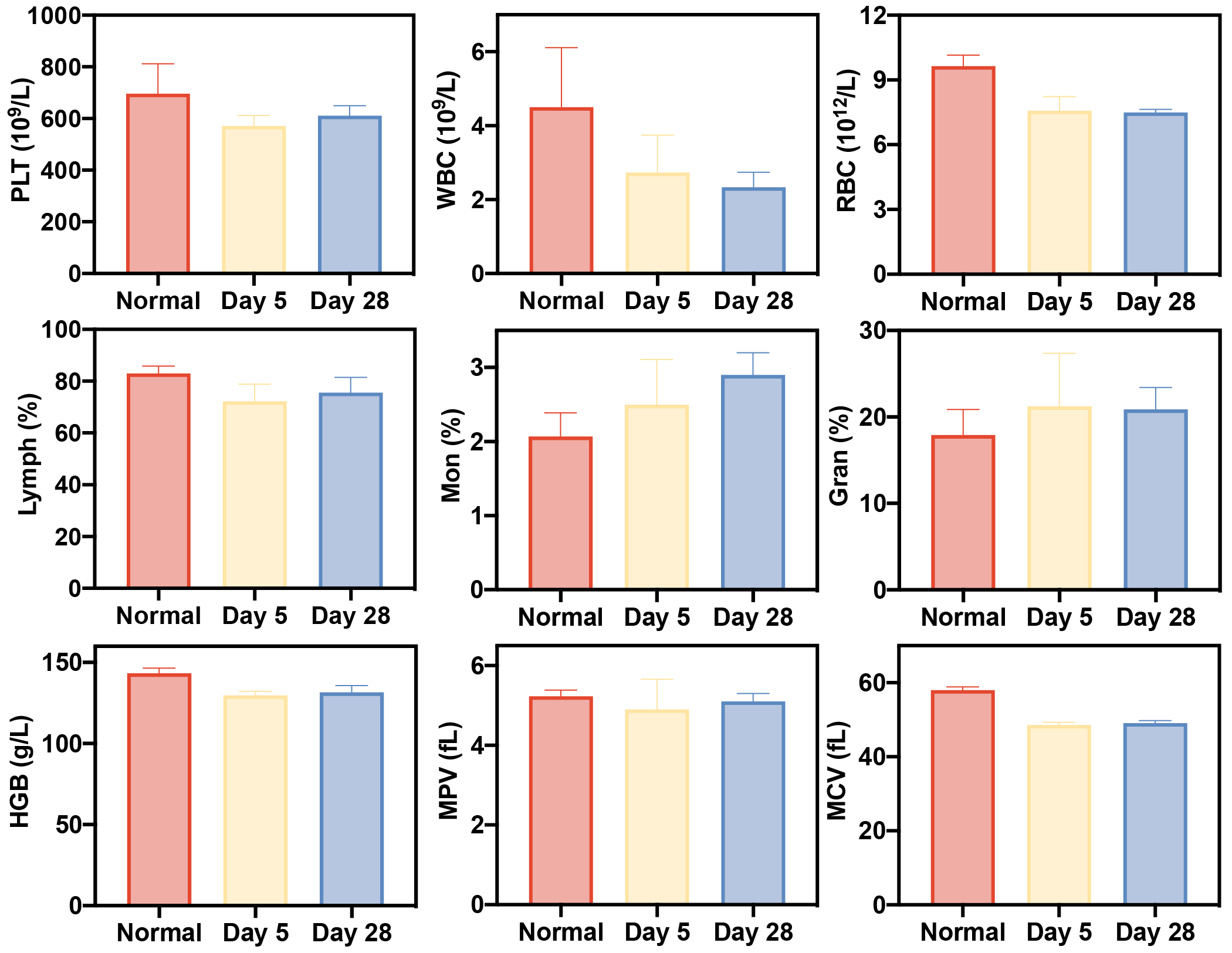


**Figure S**24**.** Hematology analysis of blood samples collected at normal mice, mice at 5 days and 28 days after intravitreal injections of 2 μL of M@CCNP (MOR: 17.8 μM, CCNP: 1 mM). Results are presented as mean ± SD, n = 3.

**
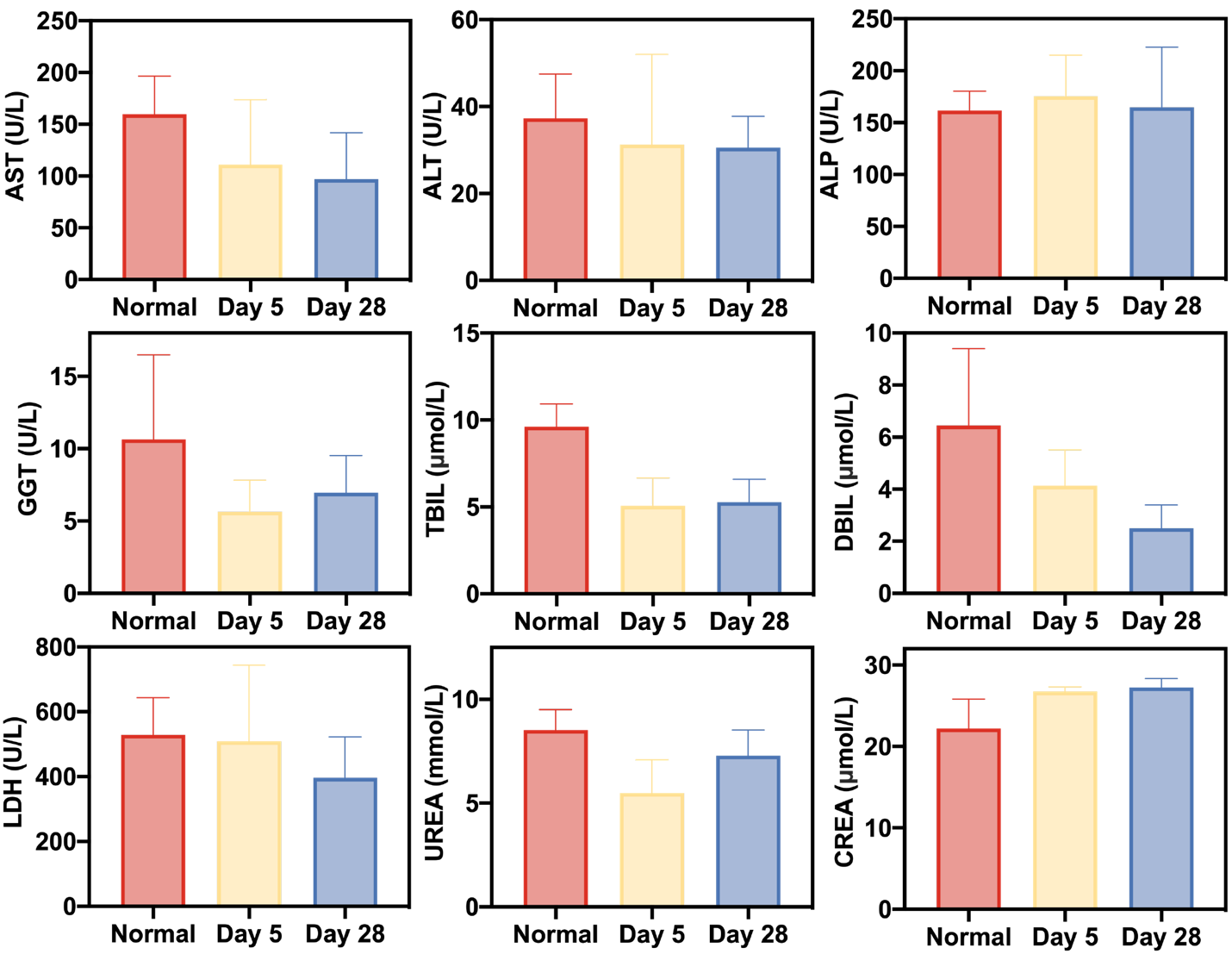
**

**Figure S**25**.** Blood chemistry analysis of blood samples collected at normal mice, mice at 5 days and 28 days after intravitreal injections of 2 μL of M@CCNP (MOR: 17.8 μM, CCNP: 1 mM). Results are presented as mean ± SD, n = 3.

**
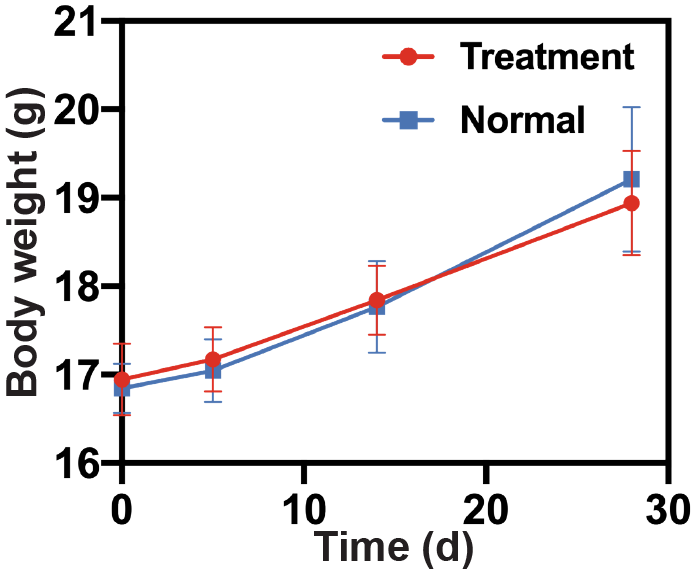
**

**Figure S**26**.** The change of body weight in 28 days of normal mice and mice after intravitreal injections of 2 μL of M@CCNP (MOR: 17.8 μM, CCNP: 1 mM). Results are presented as mean ± SD, n = 6.

**
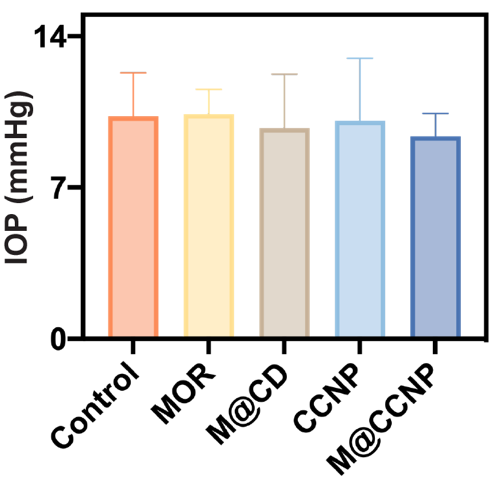
**

**Figure S**27**.** The intraocular pressure of CNV mice after varied treatment. Results are presented as mean ± SD; n ≥ 5; ns, p > 0.05; *p < 0.05; **p < 0.01.

**
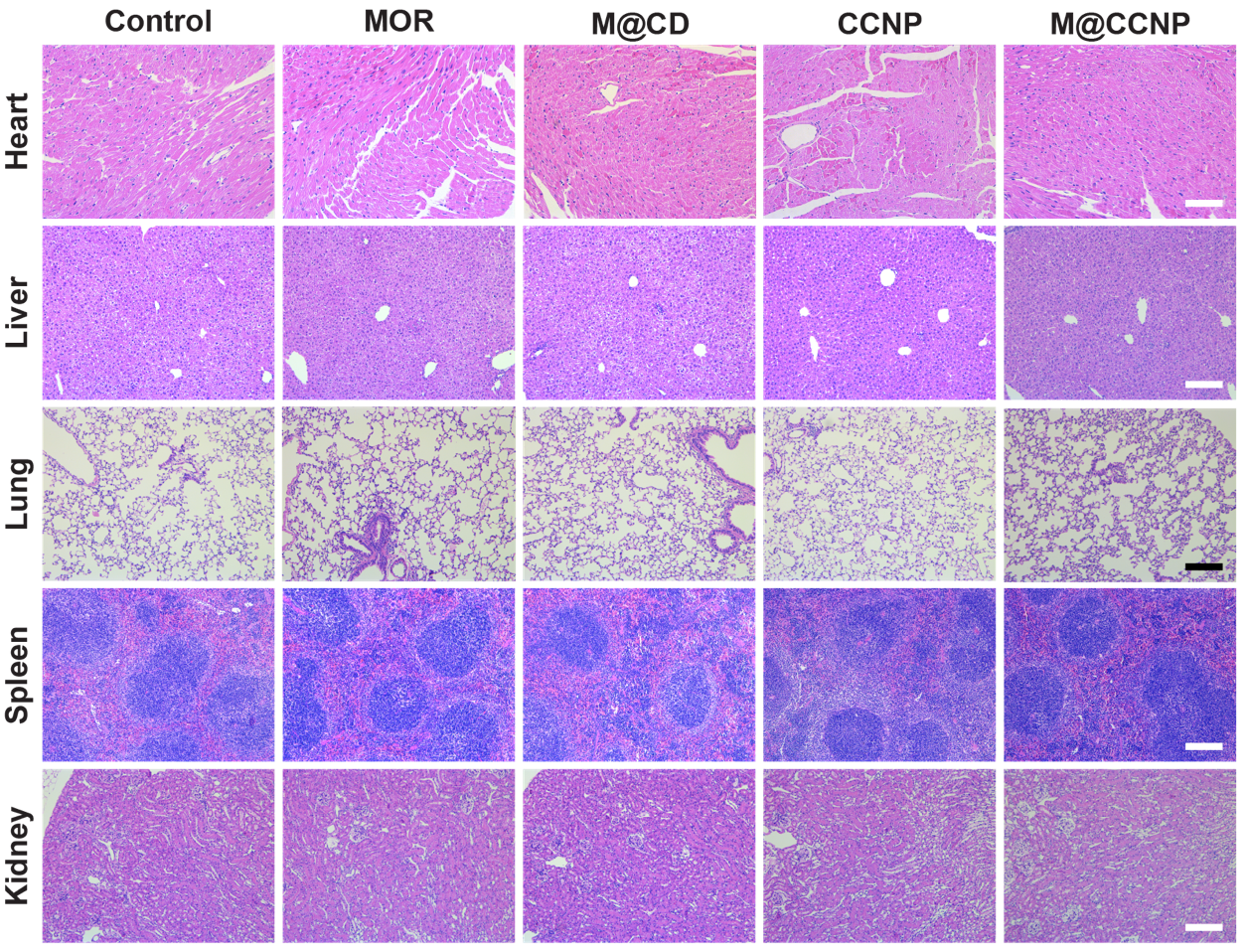
**

**Figure S**28**.** H&E staining of tissue sections of heart, liver, spleen, kidney, and lung samples collected from CNV mice after varied treatment; scale bar = 100 μm (heart)/200 μm (liver, lung, spleen, and kidney).

**
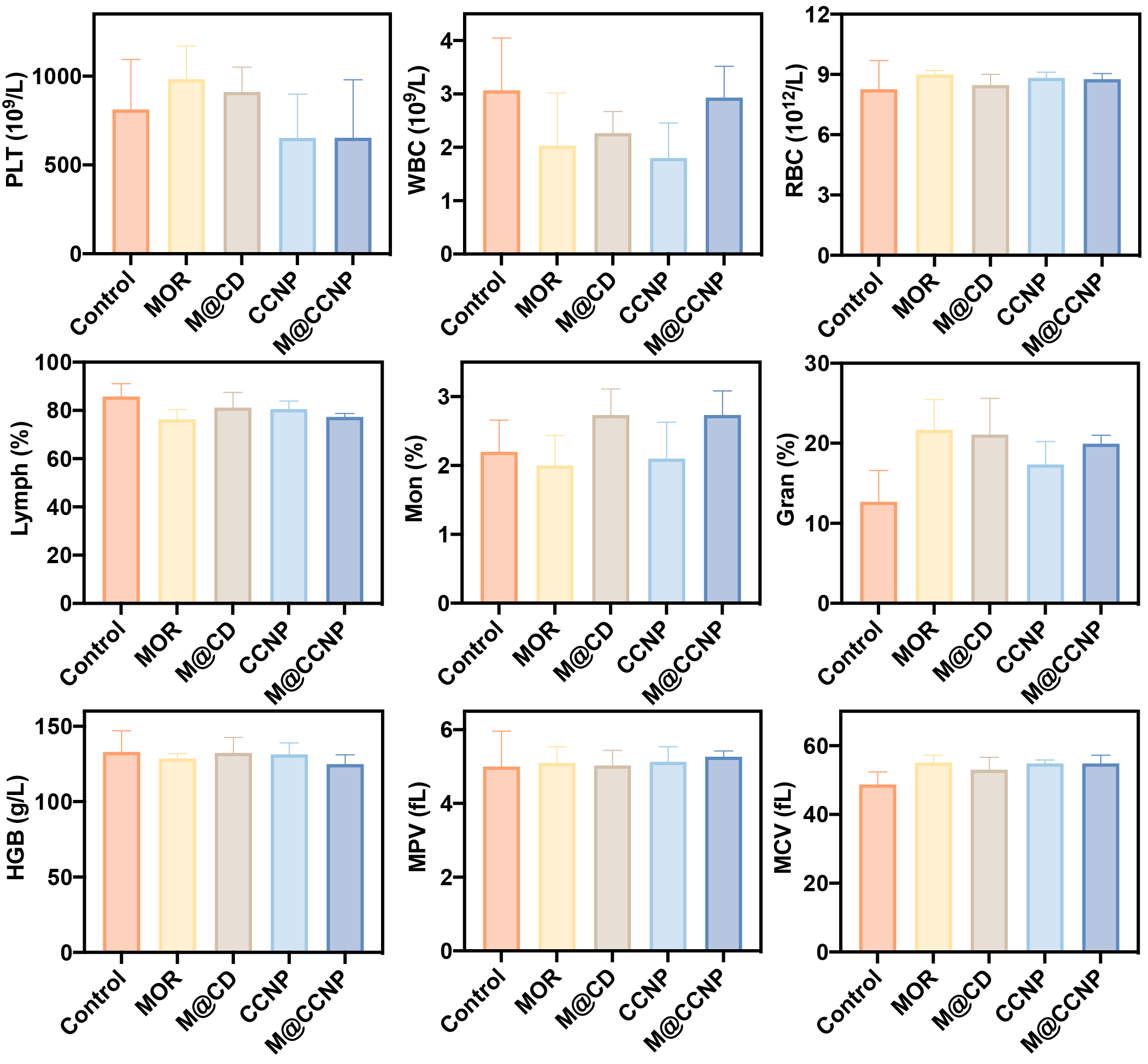
**

**Figure S**29**.** Hematology analysis of blood samples collected from CNV mice after varied treatment. Results are presented as mean ± SD, n = 3.

**
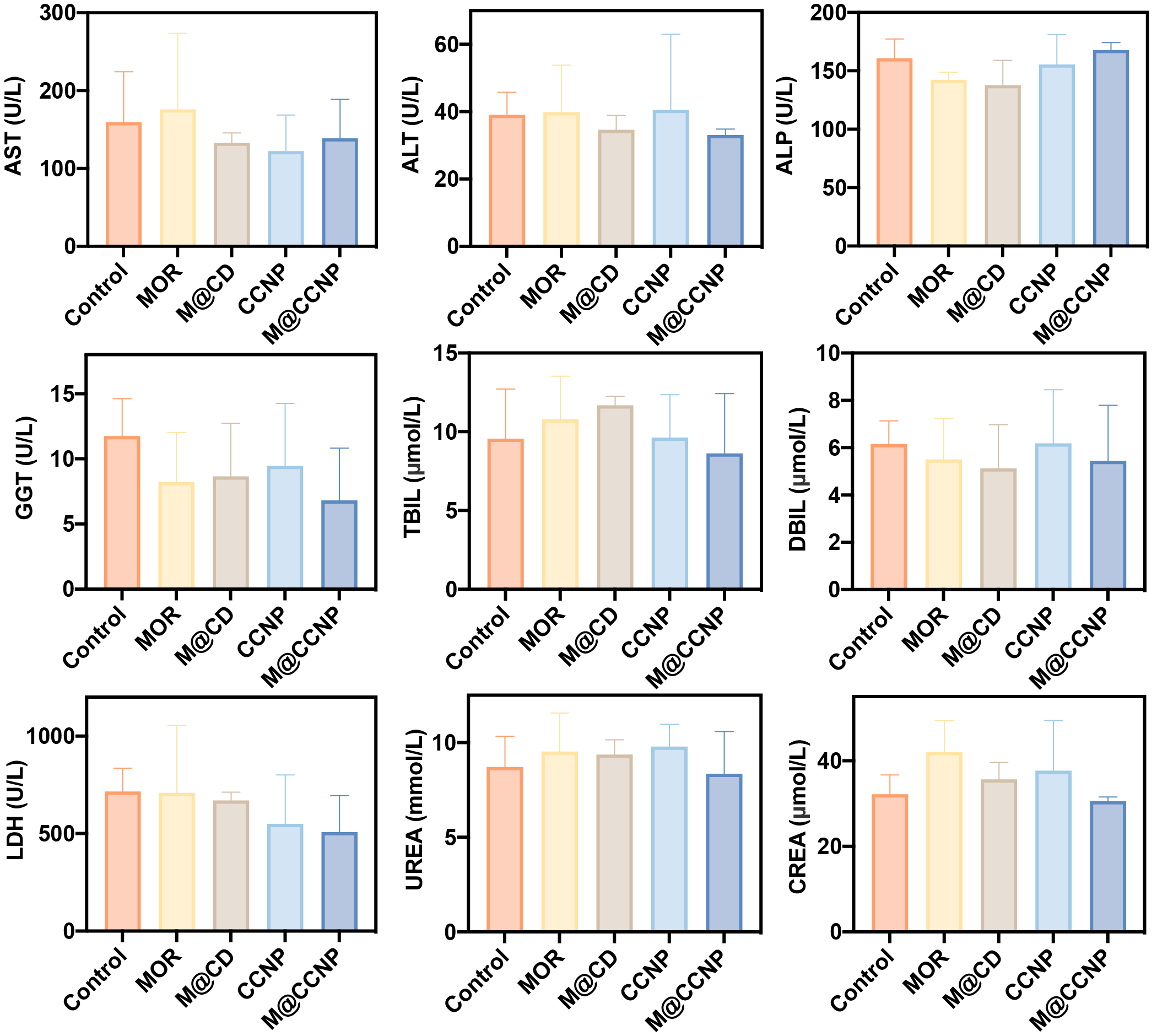
**

**Figure S**30**.** Blood chemistry analysis of blood samples collected from CNV mice after varied treatment. Results are presented as mean ± SD, n = 3.


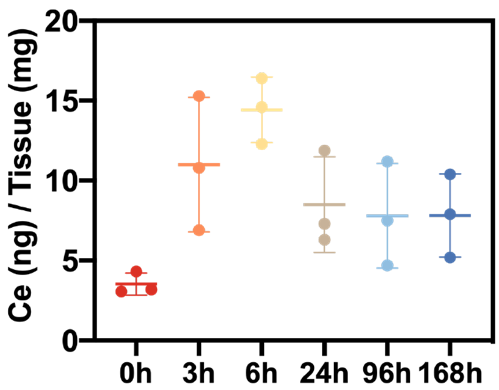


**Figure S**31. Cerium concentration in fundus post intravitreal injection of 2 uL of M@CCNP (MOR: 17.8 μM, CCNP: 1 mM) at different time points.


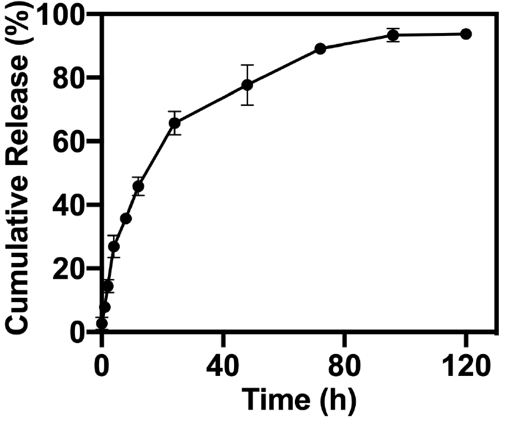


**Figure S**32. *In vitro* release profile of fluorescein from Fluorescein @CCNP in releasing medium (PBS with 0.5% (v/v) Tween-80, pH 7.4). Results are presented as mean ± SD; n = 3.

**
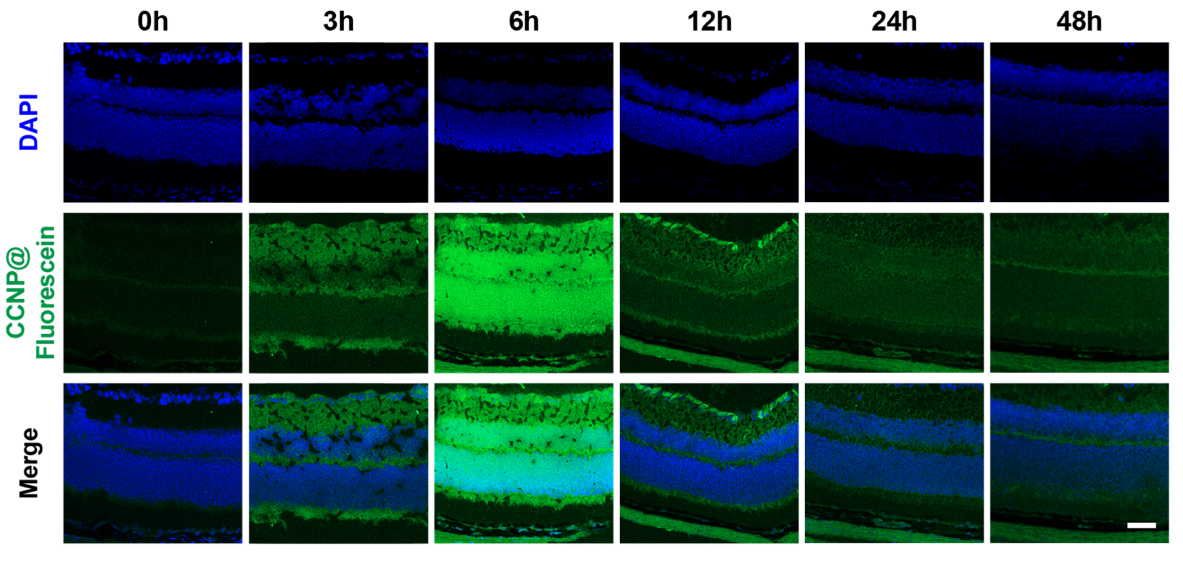
**

**Figure S**33. Representative fluorescent images of the fundus at varied time points post intravitreal injection 2 μL of Fluorescein@CCNP. Green, Fluorescein@CCNP; blue, DAPI; scale bar = 50 μm.

**
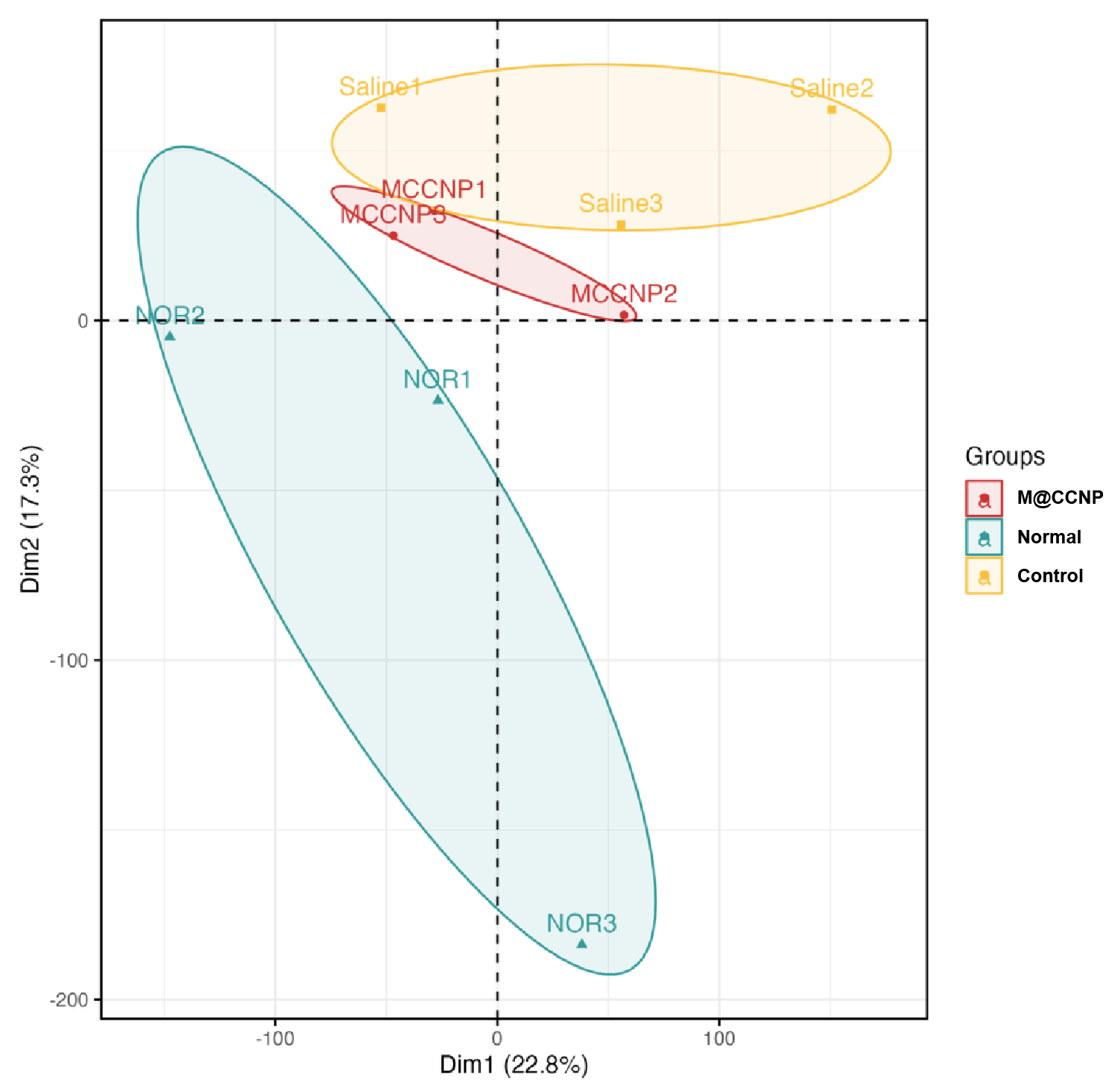
**

**Figure S**34**.** The principal component analysis (PCA) of each choroidal sample from normal mice, CNV mice with intravitreal injection of 2 μL of saline and M@CCNP (MOR: 17.8 μM, CCNP: 1 mM); n = 3.

**
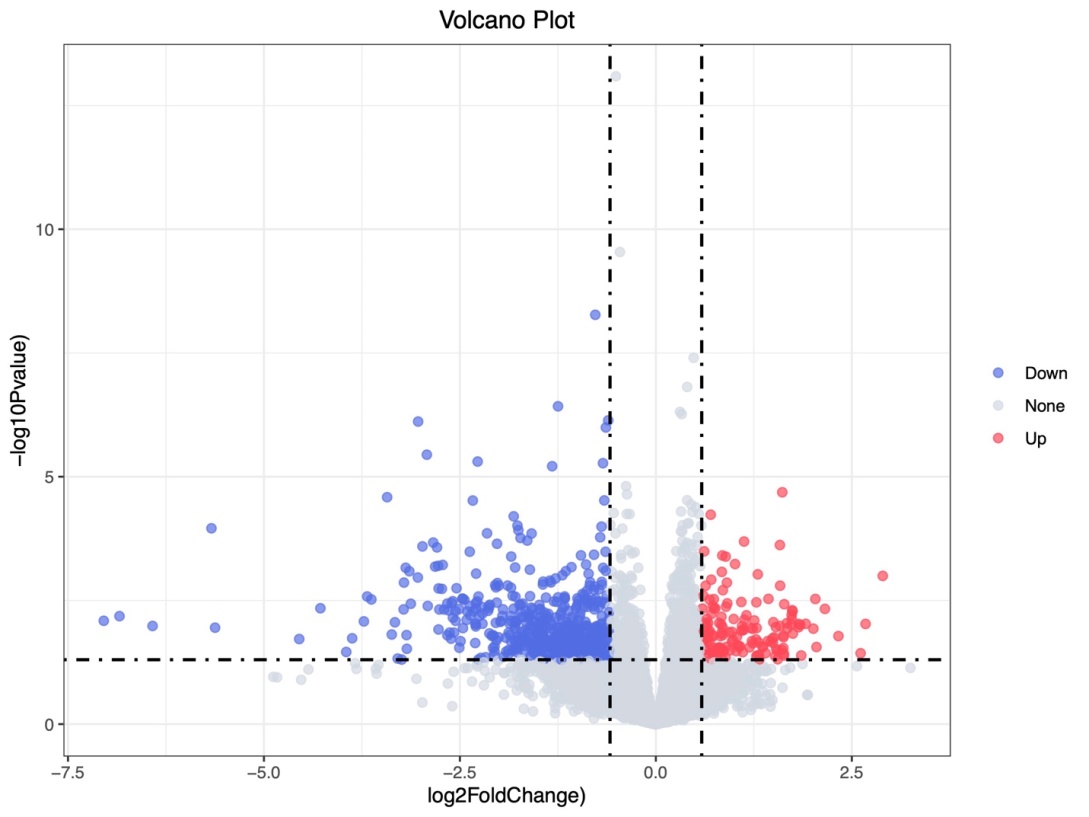
**

**Figure S**35**.** The volcano plot of the overview of the differentially expressed genes between choroidal samples from the control and M@CCNP groups. Down, downregulated genes in M@CCNP groups; None, non-differentially expressed genes; Up, upregulated genes in M@CCNP groups. Differentially expressed genes were defined as the absolute values of fold changes were equal to or greater than 1.5.

**
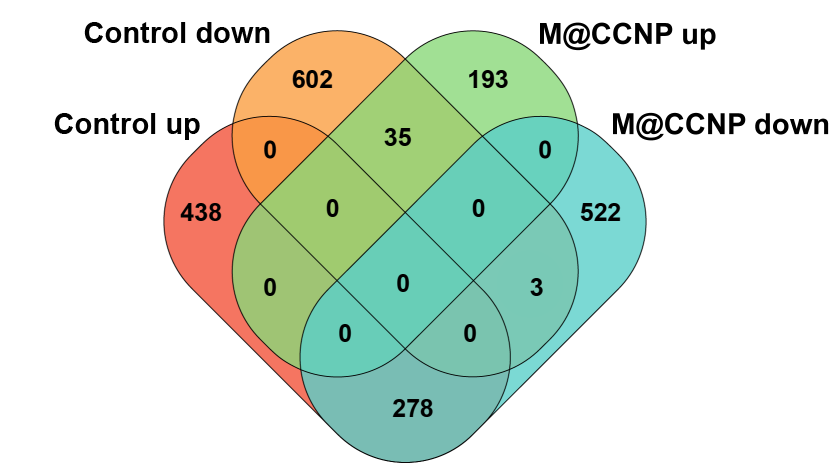
**

**Figure S**36**.** The Venn plot of the intersection set of the differentially expressed genes between choroidal samples from saline and M@CCNP groups.

**
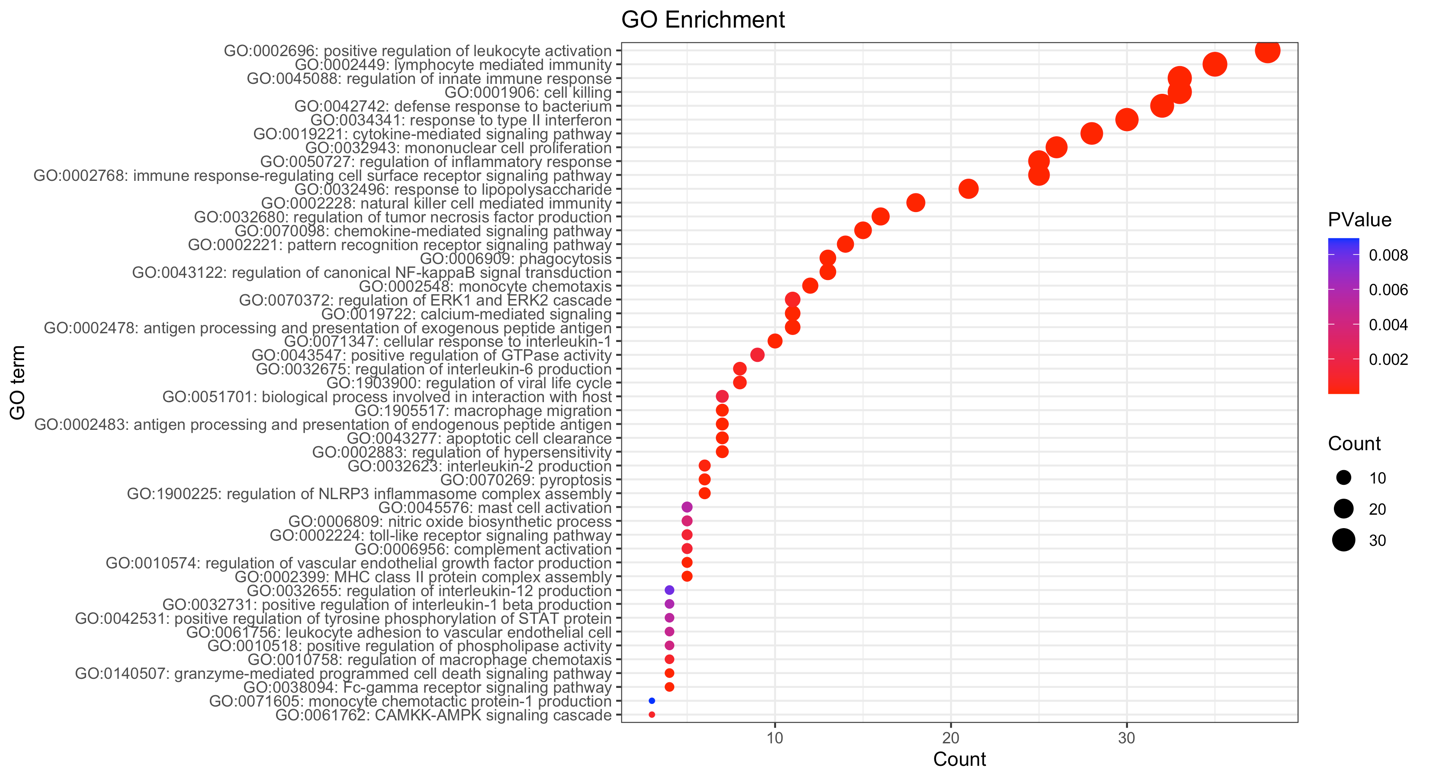
**

**Figure S**37**.** The GO pathway enrichment analysis of the differentially expressed genes between choroidal samples from the control and M@CCNP groups. Main enriched GO terms with significance were shown.


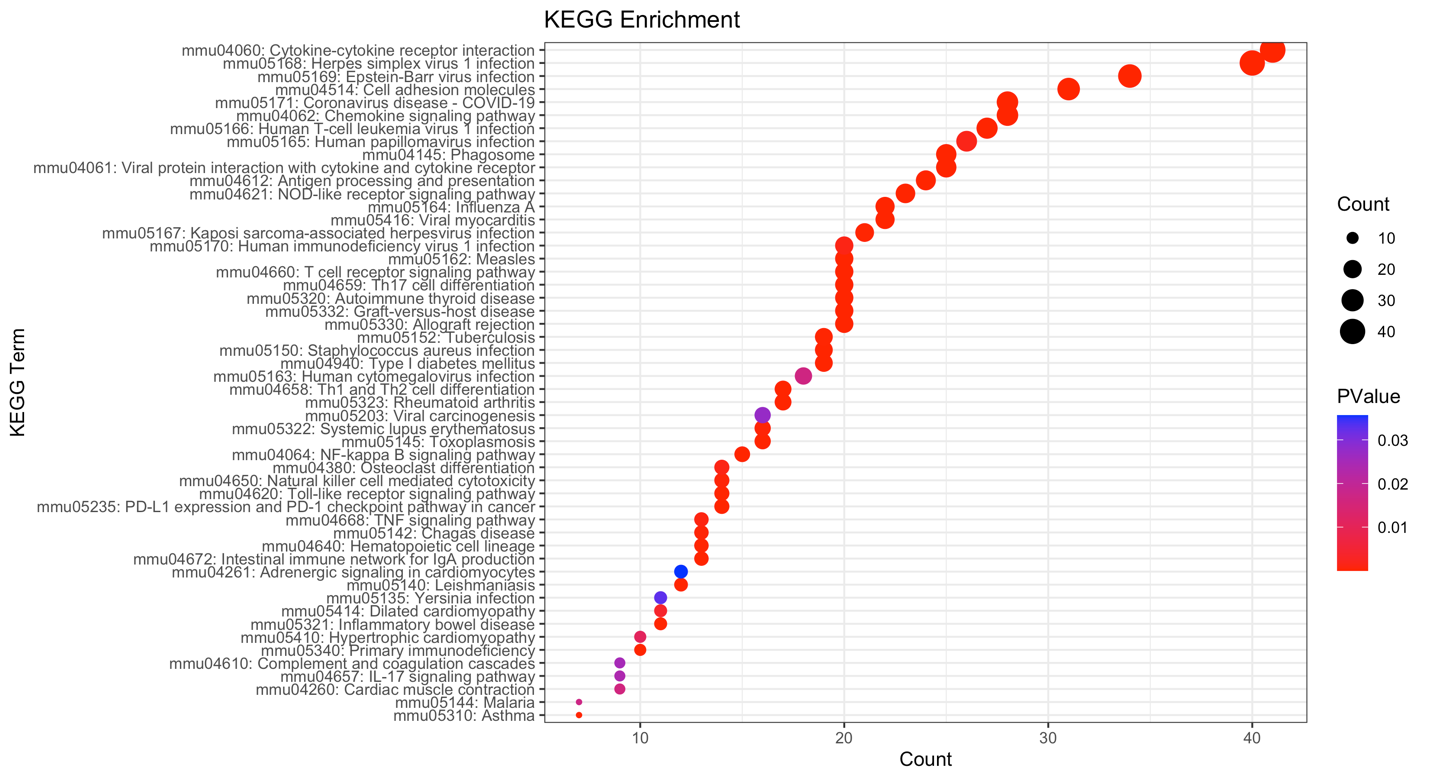


**Figure S**38. The KEGG pathway enrichment analysis of the differentially expressed genes between choroidal samples from the control and M@CCNP groups. Main enriched KEGG terms with significance were shown.

**
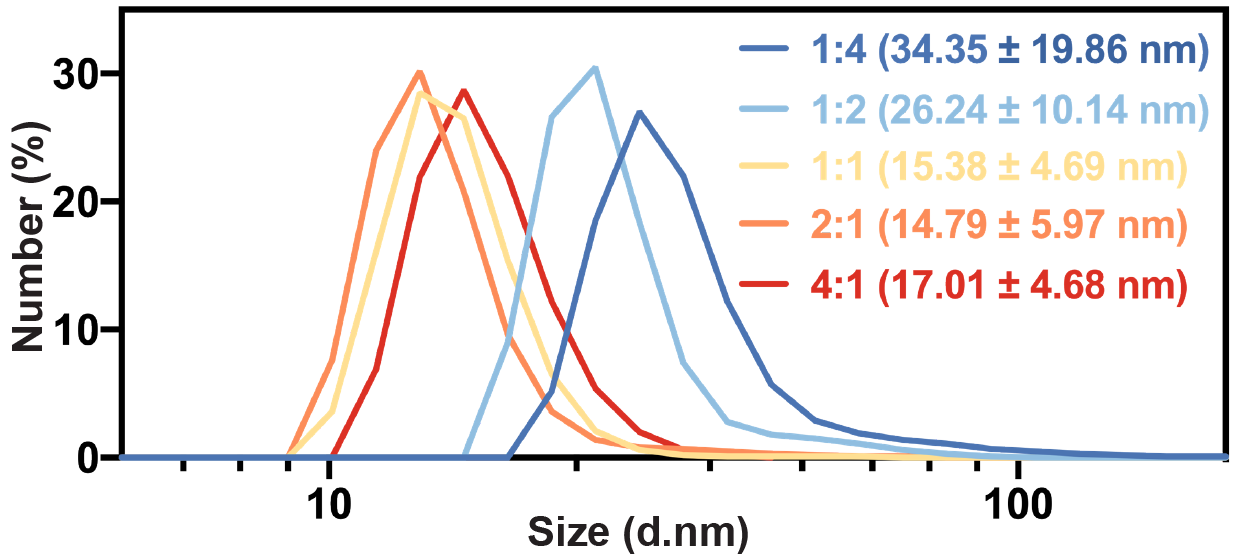
**

**Figure S**39**.** The hydrodynamic size of the final products with varied α-CD/Ce molar ratios. Results are presented as mean ± SD.


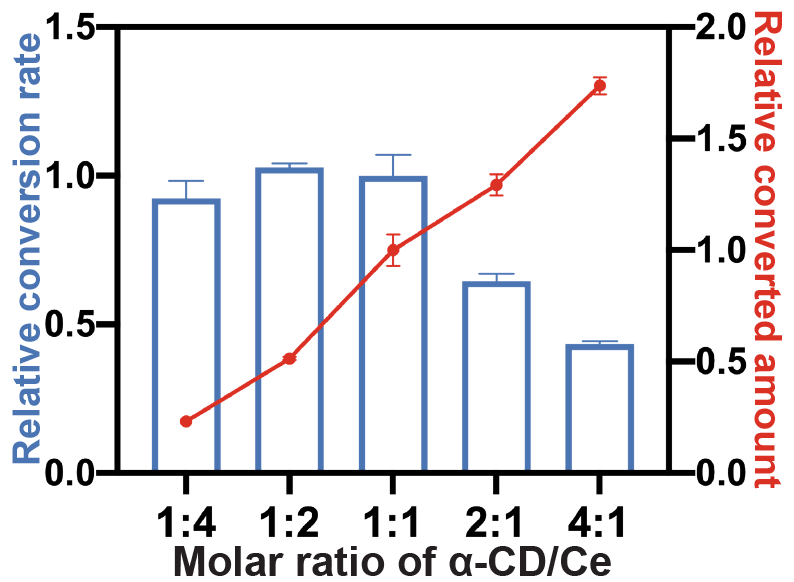


**Figure S**40**.** The relative converted amount and conversion rate of α-CD from the final products with varied α-CD/Ce molar ratios. Results are presented as mean ± SD, n = 3.

**
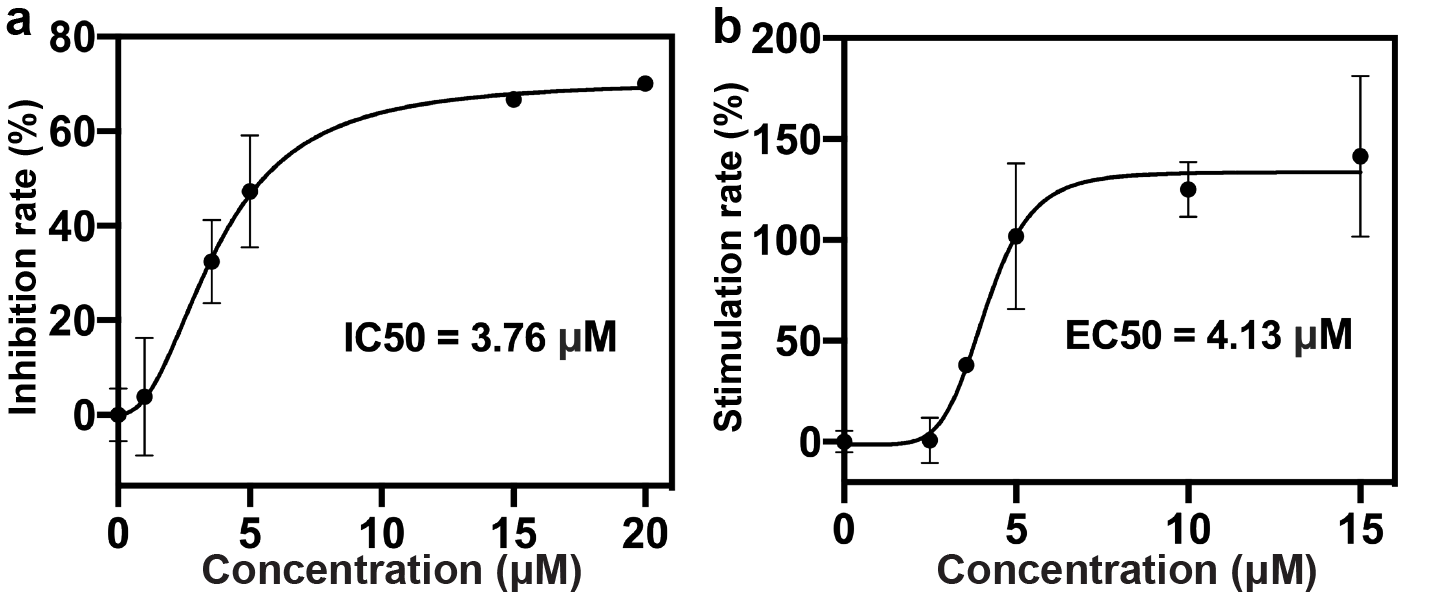
**

**Figure S**41**.** The inhibition rate of TNF-α expression (a) and the stimulation rate of NRF2 expression (b) of LPS induced RAW264.7 treated with varied MOR concentration. Results are presented as mean ± SD; n = 3.
